# The burden of disease and injury in Iran 2003

**DOI:** 10.1186/1478-7954-7-9

**Published:** 2009-06-15

**Authors:** Mohsen Naghavi, Farid Abolhassani, Farshad Pourmalek, Moradi Lakeh, Nahid Jafari, Sanaz Vaseghi, Niloufar Mahdavi Hezaveh, Hossein Kazemeini

**Affiliations:** 1 Health Programs Management Bureau, Health Network Development Center, Health Deputy, Ministry of Health and Medical Education, Hafez Jomhouri Crossroad, Tehran, Iran; 2 Currently Associate Professor of Global Health, Institute for Health Metrics and Evaluation, University of Washington, Seattle, Washington, USA; 3 Internal Diseases Department, Faculty of Medicine, Tehran University of Medical Sciences, Keshavarz Boulevard, Tehran, Iran; 4 Epidemiology and Biostatistics Department, Faculty of Health, Tehran University of Medical Sciences, Keshavarz Boulevard, Tehran, Iran; 5 Currently Post-Graduate Fellow in Global Health, Institute for Health Metrics and Evaluation, University of Washington, Seattle, Washington, USA; 6 Community Medicine Department, Faculty of Medicine, Iran University of Medical Sciences, Hemmat Expressway, Tehran, Iran; 7 Psychosocial Health Bureau, Mental Health Department, Health Deputy, Ministry of Health and Medical Education, Hafez Jomhouri Crossroad, Tehran, Iran; 8 Shaheed Beheshti University of Medical Sciences, Tehran, Iran

## Abstract

**Background:**

The objective of this study was to estimate the burden of disease and injury in Iran for the year 2003, using Disability-Adjusted Life Years (DALYs) at the national level and for six selected provinces.

**Methods:**

Methods developed by the World Health Organization for National Burden of Disease (NBD) studies were applied to estimate disease and injury incidence for the calculation of Years of Life Lost due to premature mortality (YLL), Years Lived with Disability (YLD), and DALYs. The following adjustments of the NBD methodology were made in this study: a revised list with 213 disease and injury causes, development of new and more specific disease modeling templates for cancers and injuries, and adjustment for dependent comorbidity. We compared the results with World Health Organization (WHO) estimates for Eastern Mediterranean Region, sub-region B in 2002.

**Results:**

We estimated that in the year 2003, there were 21,572 DALYs due to all diseases and injuries per 100,000 Iranian people of all ages and both sexes. From this total number of DALYs, 62% were due to disability premature deaths (YLD) and 38% were due to premature deaths (YLL); 58% were due to noncommunicable diseases, 28% – to injuries, and 14% – to communicable, maternal, perinatal, and nutritional conditions. Fifty-three percent of the total number of 14.349 million DALYs in Iran were in males, with 36.5% of the total due to intentional and unintentional injuries, 15% due to mental and behavioral disorders, and 10% due to circulatory system diseases; and 47% of DALYs were in females, with 18% of the total due to mental and behavioral disorders, 18% due to intentional and unintentional injuries, and 12% due to circulatory system diseases. The disease and injury causes leading to the highest number of DALYs in males were road traffic accidents (1.071 million), natural disasters (548 thousand), opioid use (510 thousand), and ischemic heart disease (434 thousand). The leading causes of DALYs in females were ischemic heart disease (438 thousand), major depressive disorder (420 thousand), natural disasters (419 thousand), and road traffic accidents (235 thousand). The burden of disease at the province level showed marked variability. DALY estimates by Iran's NBD study were higher than those for EMR-B by WHO.

**Conclusion:**

The health and disease profile in Iran has made the transition from the dominance of communicable diseases to that of noncommunicable diseases and road traffic injuries. NBD results are to be used in health program planning, research, and resource allocation and generation policies and practices.

## Background

This paper presents the main findings of the first National Burden of Disease (NBD) study in Iran, which was conducted for the year 2003 by the Health Programs Management Bureau of the Ministry of Health and Medical Education (MOHME) in collaboration with other departments of the Health Deputy of MOHME and Universities of Medical Sciences, with the support of the Eastern Mediterranean Regional Office (EMRO) of the World Health Organization (WHO), and senior consultancy by Professor Alan D. Lopez, Head of the School of Population Health at the University Queensland. We adopted the methodology described by WHO for conducting NBD studies to calculate Years of Life Lost due to premature mortality (YLL), Years Lived with Disability (YLD), and Disability-Adjusted Life Years (DALYs) for more than 200 disease and injury causes and 27 risk factors, as well as Health-Adjusted Life Expectancy (HALE), for the year 2003 [1]. DALYs were estimated at the national level as well as for six selected provinces individually. The results of healthy life expectancy and burden of risk factors estimation are presented elsewhere.

The history of the first Iranian National Burden of Disease, Risk Factors, and Healthy Life Expectancy (IRNBD) study goes back to a preliminary study of the burden of disease (BOD) in three provinces in 2002. The literature on burden of disease and other countries' experiences with national burden of disease studies, as well as the dialogue and advocacy about BOD in scientific and policy circles in Iran, resulted in the inclusion of these results in health policy making, as reflected in the "Law of the Fourth Economic, Social, and Cultural Development Plan of the Islamic Republic of Iran" [2]. Using the available human resources and data, and developing other necessary capacities, the National Burden of Disease Study in Iran began in 2003 and continued until late 2005. The study's team included more than fifty epidemiologists, demographers, and social medicine and clinical specialists, as well as more than ten collaborating research centers and organizations. The study's methodology, protocol, and results were published in Farsi and shared with stakeholders through presentations and publications [3].

Summary Measures of Population Health (SMPH) that describe health gaps and health expectancies reflect the attempt to measure health and make WHO's definition of health which was introduced in the 40 s operationally measurable and comparable in a global context. As populations go through the demographic, epidemiologic, and health transitions, conventional mortality measures progressively fail to fully reflect the quantity of ill health and its impact on population health. One major cause for this is the shift of populations' disease profile from the dominance of communicable, maternal, perinatal, and nutritional conditions to noncommunicable diseases, which lead to more non-fatal health outcomes as compared with their contribution to mortality. New Summary National Burden of Disease studies, which describe the level of population health with HALE, and the burden of disease with DALYs form part of a broader framework that aims at Health System Performance Assessment (HSPA) [4]. In addition to the level of population health (HALE) and health problems (DALYs), the equity in the distribution of population heath has been estimated in the HSPA framework for Iran in a collaborative study by MOHME, WHO, and the Epidemiology and Biostatistics Department, School of Public Health, Tehran University of Medical Sciences [5].

The overall objective of the first National Burden of Disease Study in Iran was to provide quantitative estimations of the burden of death and disability, their determinants, and the average level of population health. The ultimate goal of the study was to provide evidence to inform health policy making, in order to guide priorities in health intervention program planning, health and biomedical research, and resource generation and expansion.

## Methods

We used the NBD methodology recommended by WHO to compute years of life lost due to premature mortality (YLL), years lived with disability (YLD), and disability-adjusted life years (DALYs); the GBD 1990 values of C = 0.1658 and β = 0.04 were used for standard age weighting, and a discount rate of R = 0.03 – for health gain in the future. C is an adjustment constant that ensures the equality of total burden in the GBD 1990 with and without age weighting, β determines the importance of age weights, and R discount rate adjusts for health gains or losses in the future [1]. Similar methods were used to estimate the disease burden for six provinces separately (Hormozgan, East Azerbaijan, Khorasan, Bushehr, Yazd, and Charmahal & Bakhtiary). These provinces were selected based on their prior capacities for organizational change within the context of the Health Sector Reform project funded by the World Bank and commissioned by the World Health Organization, and also on their various geographical locations in Iran with different levels of socioeconomic and health development. Data sources and adjustments to the NBD methods are described below. Specific departures from the GBD methodology are presented first, i.e. cause list expansion, cancer burden estimation, comorbidity adjustment for injuries, disability weights derivation, and development of BOD management software.

### Choosing the diseases

We modified the GBD list of diseases, taking into consideration Iran's epidemiological circumstances for inclusion and exclusion of detailed disease causes. For instance, more than ten diseases (like yellow fever and trypanosomiasis) that do not exist in Iran were excluded and a few diseases of epidemiological or social importance in the country (e.g. thalassemia and brucellosis) were added to the list. The list was finally ordered by ICD-10 codes [6]. Systematic discussion with health policy makers, high-level managers, researchers, and clinical specialists ensured that no disease or injury cause with epidemiological, economic, social, political, or clinical importance at the population level was missed from the list. The detailed list of causes selected for the IRNBD is provided in the Additional file 1.

### Disease-specific modeling templates

Cancers and injuries were modeled with disease-specific templates (described below). Three other disease groups, namely perinatal and maternal conditions, and congenital malformations and chromosomal abnormalities were also modeled without using the DISMOD software. Most of the perinatal conditions, congenital malformations and chromosomal abnormalities manifest at birth and need early treatment or correction. They lead to high mortality or lifetime disabilities and do not occur during any other period of life. Maternal conditions have similar characteristics and only occur during pregnancy or shortly after delivery. In fact, the dominant measure of their frequency is incidence, whereas it is reasonable to use prevalence to measure their long-term complications.

#### Cancer

Given the incompleteness of cancer survival information, the model described by Mathers et al. [7] was used to model the cancer survival and complete the parameters needed for the epidemiological modeling of cancer and assessing their internal consistency, using the cancer registry and death registry information. A specific disease modeling template software, called "CANMOD", was developed for cancers, based on specific mortality rates from both the cancer and death registries in 2003, as well as on other sources of national and international information on cancer prevalence, incidence, and survival. This disease-specific template set the internal consistency for information from the above two sources of mortality rates along with cancer survival rates. Incidence and survival rates for cancer patients were obtained using the age-specific incidence rates estimated based on survival and mortality rates from the cancer registry. The obtained incidence rates were used for the calculation of incidence-to-mortality ratios. If these ratios were not compatible with rates deemed plausible for Iran's setting, the survival rates were gradually changed to reach such plausibility. Survival information was fitted to a Weibull distribution for all ages. Using the available data, the proportions of patients entering each possible state in the natural history of different types of cancers were calculated (i.e. A1. Diagnosed and under treatment, A21. Controlled, A22. Treated premetatstatic, B1. Untreated premetatstatic, B2. Metastatic, B3. Terminal). Then using the incidence rate, the number of patients entering each state was obtained. Using the average duration and disability weight of each state, YLDs were calculated for all states and the results summed up for that specific cancer. For assessing the internal validity of the model for each cancer, the incidence rate obtained from the model was checked against the incidence rate from the cancer registry. Given the higher coverage of the death registry as compared with the cancer registry, the model was valid if the model-based incidence was higher than the cancer registry-based incidence. The resulting burden was also compared with the burden estimated for EMR-B and the GBD for assessing the external validity of estimates.

#### Injuries

A specific model was developed for calculating the disability weight of multiple trauma patients. Individual-level data for 13,400 hospitalized trauma patients from twelve provinces studied in July to October 2003, covering a population of 9.6 million [8] were used to calculate the YLL and YLD, along with the disability weight taking into account the comorbidity. Disability durations and weights were adopted from the GBD and Victorian BOD studies [9,10]. In patients who suffered an accident and in fact the "coincidence" of multiple simultaneous injuries, comorbid disability weights were calculated through the following steps for each individual: (A) the injuries were rank ordered according to their ascending value of duration; (B) a "common disability weight" for ***i ***coincident injuries was calculated using the following formula, which is the general formula for the multiplicative model:



where DW_1 _through DW*i *are the disability weights for the longest through shortest duration injuries; (C) then for the time interval in which the injury of shortest duration had healed but the other two continued to coexist, another common disability weight was calculated as:



The above two formulae are adopted from the formulae used for correction for independent comorbidity between major condition groups in calculation of prevalence-based YLD [1,4]. (D) And for the last time interval in which only the longest duration injury continued to exist, only the disability weight of this injury, "Common DW _(1,1)_" = DW _(1)_, was taken into account. In situations where more than three injuries had occurred at the same time, a similar calculation method was used. (E) Eventually, the time interval with three coexistent injuries was multiplied by "Common DW _(1,3)_," the time interval with two concurrent injuries was multiplied by "Common DW _(1,2)_," the last time interval with only the injury with the longest duration was multiplied by "Common DW _(1,1)_" = DW _(1)_, and YLD was summed for the whole time interval with multiple injuries. The maximum number of comorbid disabilities in this template was five, since patients with six or more concurrent disabilities were extremely rare. Accidents and injuries not leading to hospitalization but resulting in emergency department care or outpatient services were included. Only the trivial injuries managed by the injured persons without receiving any healthcare services were not included. We assumed that the YLD rates for multiple trauma derived from this sample of 13,400 patients from twelve provinces studied for four months of the year 2003 were generalizable to the whole population of Iran in that year, since the variability of these YLD rates should have been low during the year and across all provinces.

### Disability weights

Disability weights for disease and injury causes in the GBD list [9] were adopted. For other disease and injury causes in our list but not in the GBD list, weights were adopted from the Dutch Disability Weights Group [11]. For 86 causes in our list and not in either the GBD or the Dutch list, we used a Delphi method for obtaining the disability weights through specialists' opinion [12]. The list of these causes, along with the GBD and Dutch lists of causes (without the disability weights) was given to a number of relevant clinical specialists (members of the specialty and subspecialty national boards). The specialists were asked to identify those causes in the GBD and Dutch lists, to which the disability profile of each of the above-mentioned 86 causes was most similar. The most frequent responses were extracted. The specialists were then asked about the disability weight that they would assign for each cause, given the summary results of the first round, and the average weights were drawn. Finally, the list of 86 causes and their mean given disability weights was presented to the respondents for the finalization of the disability weights, taking into consideration the average weights, their own individual responses, and their clinical judgment about the similarity of disability profiles. Furthermore, the respondents were asked to identify any sex and age differences in weights, if applicable. Disability weights for all causes in the GBD and IRNBD studies are provided [see Additional file 2].

### Burden of disease management software

There were 213 disease and injury causes for which epidemiological disease modeling was performed, each of these causes had more than five spreadsheet files on average, and there were many other spreadsheet files based on templates for YLL and DALY calculations. Thus, a "Burden of Disease Management" software using Access and Excel was developed to contain, relate, and manage all the input data, intermediate calculations, and output results for DALY estimation. This software also contained the prevalence-based YLD estimation sheets, which were used for the estimation of healthy life expectancy.

### Population estimation

The population of Iran in 2003 was estimated by age group and sex (using the last census from 1996). The following formula was used for estimating the population at age x in each sex separately:



in which P_(x+1, t+1) _is population at age *(x+1) *in one year after the reference year, P_(x, t) _is population at age *x *in year *t *or the reference year, S_x _is the survival probability from age *x *to *(x+1)*, I_x _is the number of immigrants of age *x*, and O_x _is the number of emigrants of age *x*. The baseline population for age zero was the number of births in year *t*. The age-specific fertility rates were multiplied by the population of women between the time intervals 1996–2001 and 2001–2006, and then multiplied by a proportion of 0.488 to obtain the number of female births and by 0.512 to obtain the number of male births. The population in five-year groups (starting from 0–4 years) was estimated using the survival probabilities from birth to six months or to 2.5 years and in a similar way for n-year age groups. In this way, populations of all age-sex groups were estimated for the reference year 2003.

### Mortality and YLL

For the estimation of mortality rates and YLL, data from the national death registry of MOHME [13] were used and garbage codes were redistributed. The death registration system of Iran's MOHME covered four provinces in 1999, and 23 out of Iran's 28 provinces in 2003 with a population of 48,379,552 (i.e. 73% of the national population of 66,518,224). Deaths and population data from these 23 provinces were used for the estimation of mortality rates, YLL, and also total life expectancy and healthy life expectancy. The system extended its coverage to 29 out of 30 provinces in 2005, and full national coverage will be achieved in 2008. Iran's death registration system has been described as "a good example" of "a system of death registration with medical information on the cause of death" [14], developed in recent years in order to capture the necessary mortality data, following the publication of the initial results of the GBD study. For Iran, District Health "Networks" deliver inpatient and outpatient health care in governmental hospitals and outpatient service delivery points, and supervise service delivery by other sectors. The District Health Department (DHD) is part of the District Health Network and is primarily responsible for preventive and primary health care services. The DHD is the most peripheral structural and functional unit of the District Health System and is responsible for gathering death data in coordination with the Civil Registration Organization. Since 1998, a single unified death certificate is being used, which is legally required to be issued by a physician for interment. This form is compatible with the ICD-10 and identifies the immediate, intermediate, and underlying causes of death for all ages, and four causes of stillbirths and deaths before the seventh day of life (maternal immediate, maternal underlying, fetal immediate, and fetal underlying causes). Since 1998, all physicians are being trained on how to fill out this form as a part of their compulsory continued medical education. In special cases, death certificates need to be issued by the Forensic Medicine Organization. DHD uses five sources of death data for death certificates: (1) district hospital(s), (2) cemeteries, (3) Forensic Medicine Organization, (4) service delivery points of DHD, and (5) complementary sources such as the clergy and voluntary health workers that become aware of any deaths. Using a software package specifically designed for this purpose, data are compiled and checked for duplicate entries at the district level, sent to the province center on a monthly basis, and finally processed at the national level for correction of any residual duplication and redistribution of garbage codes for cause of death. The primary criterion for registration of death locality is the place of usual residence. The software does not permit entry of impossible causes of death (like maternal causes for males) and prompts for investigation of improbable causes (like maternal causes in females younger than ten years), by checking hospital records or verbal autopsy information [13]. The mechanism for mortality data quality assurance involves a number of factors and measures. These factors which are incorporated in the death registration system of MOHME include: (1) Using multiple sources of data to minimize missing of deaths, (2) Establishment of a uniform death certificate throughout the country, (3) Provision of the necessary training for the assignment of one correct cause to a case of death, (4) Provision of training and guidelines for the assignment of a cause of death to a disease or injury cause using the ICD list of causes modified for the epidemiological situation of Iran, and (5) Preventing the assignment of garbage codes to death cases. The training of physicians on correct completion of death certificates is crucial for achieving goals 3 to 5 above [13]. The sources of population estimates for the death data system for urban areas were the baseline population of the 1996 census, population growth rate resultant from comparison of the 1986 and 1996 censuses, and adoption of the arithmetic progression intercensal method, at the district and province levels. In rural areas, annual census conducted by health houses and mobile outreach teams of the health network provide the population data. Eleven garbage codes for cause of death were redistributed to the registered causes in the same age-sex group, within the same ICD-10 chapter or in other chapters depending on the nature of the garbage code. For example, "heart failure" was redistributed among some of the circulatory and respiratory disease chapters, or "other ill-defined and unknown causes of mortality" was redistributed among all causes. Table 1 shows the redistribution methods for the eleven garbage codes and the relative frequency of these codes. The proportion of garbage codes in Iran's death registry was 17%, 20%, and 24% of the total deaths in 2003, 2002, and 2001 respectively [13]. The two assumptions, stable population and no migration, required for the Brass Growth-Balance and Bennett-Horiuchi methods of death under-registration correction [1] did not hold true for Iran's population. Its total fertility rate dropped from 5.5 in 1990 to 2 in 2003 [15]. We applied the Bennett-Horiuchi method using the 1996 and 2006 census data, and the resultant values of the Synthetic Extinct Generation were 1.35 to 1.05 for males between the ages of 0 and 75, and 1.20 to 1.05 for females in the same age range. This problem was traced to originate from the population by age figures from the above-mentioned censuses. The 0–4 year age group not only had no deaths during the intercensal years, but also experienced a 550 thousand increase according to census data. Similar problems were found with other age groups. Instead, we used death data from the Civil Registration Organization for estimation of under-registration in MOHME's death registration system.

**Table 1:** Redistribution methods for garbage codes of death, Iran 2003

**Garbage code** (% all causes) *(% garbage codes)*	**Redistribution method**
Senility (8%) *(49%)*	Within each age group above 59 years and in both sexes, redistributed among 5 following groups proportional to size of registered deaths: (1) Infectious and parasitic diseases: tuberculosis (pulmonary and extrapulmonary), other diarrheal disease, typhoid fever, other intestinal infections, viral hepatitis, sexually transmitted diseases, viral hemorrhagic fevers, hydatid cyst, anthrax, other infectious and parasitic diseases; (2) Diseases of the blood and blood-forming organs and certain disorders involving the immune mechanism: anemias, other diseases of blood and blood-forming organs; (3) Unintentional injuries: all causes except transport accidents, burns, and drowning; (4) Intentional self-harm: only suicides with chemicals, drugs, opium, and homicide; (5) All causes within the following chapters: neoplasms; endocrine, nutritional and metabolic diseases; mental and behavioral disorders; diseases of the nervous system; diseases of the circulatory system; diseases of the respiratory system; diseases of the digestive system; diseases of the skin and subcutaneous tissue; diseases of the musculoskeletal system and connective tissue; and diseases of the genitourinary system. Death registration software does not permit entry of senility for persons younger than 60 years of age.

Unspecified causes of death (4%) *(27%)*	Within each age and sex group, redistributed among registered causes proportional to size of registered deaths for each cause.

Heart failure (1%) *(8%)*	Within each age and sex group, redistributed between 2 ICD-10 chapters proportional to size of their registered deaths: (1) Some diseases of the circulatory system, and (2) Some diseases of the respiratory system.

'Under investigation' (0.7%) *(4%)*	Within each age and sex group, redistributed among registered causes proportional to size of registered deaths for each cause.

Septicemia (0.5%) *(3%)*	Within each age and sex group, redistributed among 6 ICD-10 chapters proportional to size of their registered deaths: (1) Infectious and parasitic diseases, (2) Some diseases of the digestive system, (3) Some of the neoplasms, (4) Some diseases of the respiratory system, (5) Some of the conditions originating in the perinatal period, and (6) Some of the causes related to pregnancy, childbirth, and the puerperium.

Unspecified neoplasms (0.5%) *(3%)*	Within each age and sex group, redistributed among the neoplasms chapter proportional to size of registered deaths for each code.

Other ill-defined and unknown causes of mortality (0.4%) *(2%)*	Within each age and sex group, redistributed among registered causes proportional to size of registered deaths for each cause.

Mental retardation (0.2%) *(1%)*	Within each age and sex group, redistributed among registered causes proportional to size of registered deaths for each cause.

Suspected homicide (0.2%) *(1%)*	Within each age and sex group, redistributed among registered causes proportional to size of registered deaths for (1) Unintentional and intentional self-harm, and (2) Assault.

Convulsions (epilepsy excluded) (0.2%) *(1%)*	Within each age and sex group, redistributed among registered causes proportional to size of registered deaths for each cause.

Sudden Infant Death Syndrome (without autopsy) (<0.001%) *(<1%)*	Within the same age group and both sexes, redistributed among all causes proportional to size of registered deaths for each code.

### Morbidity and YLD

For the estimation of disease incidence rates and YLD, epidemiological disease modeling was performed for 213 disease and injury causes, mostly using the DISMOD II software [16]. For a number of disease groups (namely injuries, perinatal conditions, congenital malformations and chromosomal abnormalities, maternal conditions, and neoplasms), epidemiological disease modeling was performed using modeling templates developed specifically for this study. Systematic reviews of all sources of data and epidemiological and clinical studies containing information on the needed inputs for disease modeling were conducted in collaboration with clinical medicine specialists. The following sources of information on the epidemiology of diseases in Iran were used for epidemiological disease modeling:

(1) Disease Surveillance System: The surveillance system has complete coverage for a number of infectious diseases such as polio, measles, and Crimean-Congo Hemorrhagic Fever. For others with incomplete coverage, e.g. HIV, malaria, and tuberculosis, estimates of surveillance system coverage were used to find the actual number of cases.

(2) Cancer Registry: Almost all sites of cancer are covered by the cancer registry system, which is based primarily on pathology reports, and therefore does not provide good coverage for sites that are not routinely biopsied, such as the central nervous system, lung, and pancreas [17]. Given the incomplete coverage for some cancer cites, a specific modeling approach described above was used for modeling cancer dynamics.

(3) Hospital Disease Registries: Hospital registries were used for diseases that require surgery, such as inguinal hernia, cholecystitis, and cholelithiasis. In most provinces, hospital disease registries for such diseases have near-complete coverage. The Iranian health services utilization study of 2002 [18] showed that with an average waiting time of three days for all inpatient needs, 90% of the cases where hospitalization was necessary resulted in hospitalization within the same province where the cases originated, while 10% were moved to a hospital in a different province. Therefore, a correction factor of 1.11 was used for data from hospital registries for such diseases. We added the cases to the provinces of origin and subtracted them from the provinces they went to.

(4) Representative National Surveys. Most surveys use structured interviews based on questionnaires, with clinical examination and paraclinical measurements where necessary, including anthropometric measurements, Decayed-Missing-Filled teeth, or blood and urine biochemistry. The main diseases in this category were mental and behavioral diseases including opioid use, malnutrition and micronutrient deficiencies including anemias, oral conditions, and some of the maternal and perinatal conditions. The most important surveys were 'Study of Health and Disease' [19], 'Profile of Population and Health: Demographic and Health Survey (DHS)' [20], 'Profile of Child Nutrition in Provinces' [21], 'National Study of Micronutrients' [22], 'Epidemiological Survey of Psychiatric Disorders in Iran' [23], 'The Epidemiological Study of Drug Abuse in Iran' [24], and 'Integrated Monitoring and Evaluation System for Reproductive Health Programs' [25].

(5) Subnational and Local Studies, results of which could be generalized to the national level, e.g. the COPCORD Study (Community Oriented Program for Control of Rheumatic Diseases) [26], Isfahan Healthy Heart Study [27], Persian Gulf Healthy Heart Study [28], Tehran Eye Study [29], and Epidemiologic Study of Injuries due to External Causes in Iran [8].

### Residual YLD estimation

Since YLLs are calculated for all registered deaths, but YLDs are not estimated for rare disease and injury causes, if the residual YLDs are not estimated, the DALY estimates will be biased towards over-representation of YLL. In comparison with the GBD disease list, our more comprehensive list of disease and injury causes already leads to lower burden left in residual areas. The residual YLD for each group was estimated using the YLD/YLL ratio for specified conditions in the group.

### Burden estimation at province level

The same methods were used for estimation of YLL, YLD, and DALY at the province level, using input data from studies many of which were applicable at the province level. The 6 selected provinces were the provinces in which the Health Sector Reform (HSR) projects of MOHME supported by WHO and the World Bank in Iran were being implemented as pilot projects, to be extended to the whole country later. These provinces where chosen based on their prior capacities for organizational change in health sector and taking into consideration the fact that they should represent the provinces with relatively higher and lower levels of socio-economic and health development.

### Regional comparisons

Burden of disease estimates from this study were compared with the revised Global Burden of Disease 2002 estimates for the Eastern Mediterranean Region (EMR) and its sub-region B (EMR-B) [30] for relative magnitude of DALY rates and rankings of the leading causes of burden.

## Results

Detailed tabulations of deaths, YLL, YLD, and DALYs for the 213 causes included in the IRNBD, classified by GBD and ICD groups, age, and gender are provided in the Additional file 1. Below we report the key findings for the burden of disease and injury (DALYs); the mortality burden due to premature deaths (YLL); and the disability burden due to non-fatal health outcomes (YLD), along with regional comparisons, and burden of disease estimates for six provinces.

### (A) Leading causes of DALYs

**Figure 1:**
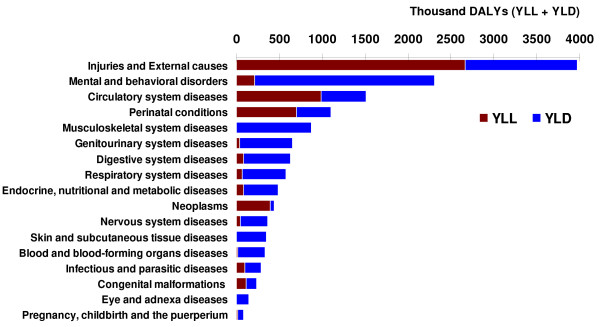
**Burden of disease groups by DALYs, all ages and both sexes, Iran 2003**.

**Figure 2:**
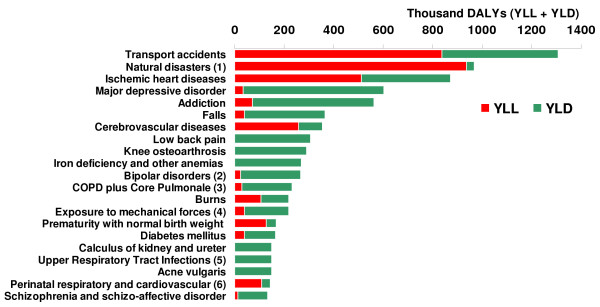
**Top 21 disease and injury causes of ICD-10 with the highest burden, all ages and both sexes, Iran 2003**. (1) Natural disasters: Exposure to forces of nature (including the Bam earthquake of 26 December 2003); (2) Bipolar disorders I, II, and cyclothymia; (3) COPD: Chronic Obstructive Pulmonary Disease; (4) Exposure to mechanical forces of the nature; (5) Upper Respiratory Infections and otitis media; (6) Respiratory and cardiovascular disorders specific to the perinatal period. Note: The rationale for presenting the 21 highest burden of disease and injury causes in this figure is that if the Bam earthquake is omitted – since such a huge disaster is not to be repeated every year – still the 20 highest burden disease and injury causes are presented.

The burden of disease and injury resulting from premature deaths and disability was estimated as a total of 14.3 million DALYs in 2003, comprised of 8.8 million years lived with disability (YLD) and 5.5 million years lost due to premature death (YLL), or 62% and 38% of total DALYs respectively. Rates per 100,000 people were 21,572 for DALYs, 13,271 for YLD, and 8,301 for YLL. Noncommunicable diseases (group II of GBD) caused 58% of the total number of DALYs, injuries (group III of GBD) caused 28%, and communicable, maternal, perinatal, and nutritional conditions (group I of GBD) caused the remaining 14%. The three disease groups causing the highest DALY rates in all ages and both sexes were injuries (28% of the total), mental and behavioral disorders (16%), and circulatory system diseases (10%) (see figures 1 and 2). Table 2 shows YLL, YLD, and DALY rates for the three GBD groups. Table 3 shows the 20 top disease groups with the highest mortality, YLL, YLD, and DALY rates for all ages and both sexes.

**Table 2:** Diseases with the highest burden; all ages and both sexes, Iran 2003

	**Measure (per 1,000)**					
	
**Disease**	**YLL rate**		**YLD rate**		**DALY rate**	
	
	**Disease**	**Value**	**Disease**	**Value**	**Disease**	**Value**
Total	All diseases	83.0	All diseases	132.7	All diseases	215.7

GBD clusters	Injuries ^(1)^	40.1	NCD^(2)^	95.1	NCD ^(2)^	125.4
	
	NCD ^(2)^	30.3	Injuries ^(1)^	19.6	Injuries ^(1)^	59.7
	
	C.M.P.N. ^(3)^	12.7	C.M.P.N. ^(3)^	17.9	C.M.P.N. ^(3)^	30.6

(1) Group III of GBD; (2) NCD: Noncommunicable Diseases (group II of GBD); (3) C.M.P.N.: Communicable, Maternal, Perinatal, and Nutritional conditions (group I of GBD)

**Table 3:** The top 10 disease and injury causes with the highest mortality, YLL, YLD and DALY rates/all ages and both sexes, Iran 2003

**Detailed causes**	**ranked by mortality rates (in 100,000)**	**Detailed causes ranked by YLL rates**	**(in 100,000)**	**Detailed causes ranked by YLD rates**	**(in 100,000)**	**Detailed causes**	**ranked by DALY rates (in 100,000)**
Ischemic heart disease	109	Natural disasters^(1)^	1409	Major depression ^(2)^	852	Road traffic injuries	1963

Natural disasters^(1)^	61	Road traffic injuries	1259	Mental disorders due to opioid use ^(3)^	735	Natural disasters ^(1)^	1455

Road traffic injuries	48	Ischemic heart disease	771	Road traffic injuries	704	Ischemic heart disease	1310

Cerebrovascular disease	45	Cerebrovascular disease	388	Ischemic heart disease	539	Major depression ^(2)^	904

Hypertension and its complications	17	Suicide	200	Falls	487	Mental disorders due to opioid use ^(3)^	844

Stomach cancer	12	Premature birth with appropriate weight	192	Low back pain	463	Falls	548

Perinatal diseases due to length of pregnancy	8	Perinatal respiratory and cardiovascular disorders ^(4)^	163	Knee osteoarthritis	438	Cerebrovascular disease	532

Diabetes Mellitus	7	Burns	162	Iron deficiency & other anemias	403	Low back pain	463

COPD ^(5)^	7	Homicide	124	Bipolar disorder	367	Knee osteoarthritis	438

Asthma	7	Premature birth with low weight	116	COPD ^(5)^	306	Iron deficiency & other anemias	405

(1) Natural disasters (exposure to forces of nature) including the Bam earthquake of 26 December 2003; (2) Major depressive disorder; (3) Mental and behavioral disorders due to use of opioids or opioids with multiple drugs; (4) Respiratory and cardiovascular disorders specific to the perinatal period; (5) COPD: Chronic obstructive Pulmonary Disease

### (B) Sex differentials for leading causes of DALYs

**Figure 3:**
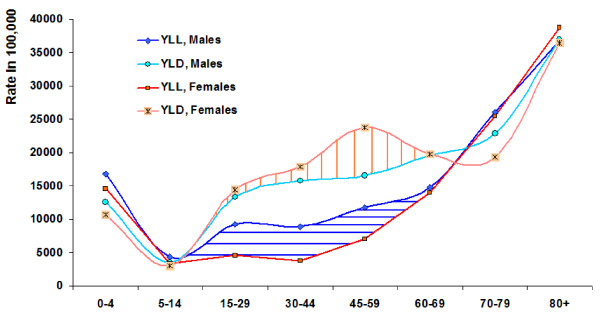
**Sex differentials in burden of disease by age, Iran 2003**. The orange shaded area under the YLD curve for females between ages 15–69 shows the extra burden of disability for this group. The blue shaded area under the YLL curve for males between ages 5–69 shows the extra burden of mortality in this group.

The burden of DALYs was higher for males (53%) than for females (47%). Sixty-one percent of YLLs and 48% of YLDs were in males, while 39% of YLLs and 52% of YLDs were in females. The DALY rate per 1,000 people was 227.1 years for males and 204.0 years for females. YLL rates were 100.4 year per 1,000 males and 65.3 years per 1,000 females. YLD rates were 126.8 and 138.8 years per 1,000 persons for males and females respectively. The burden of disability was higher in females, while males suffered from a higher burden of premature death, which led to more burden of disease and death altogether for men as compared with women (see figure 3). Table 4 shows the top 20 ICD-10 disease and injury causes with the highest DALYs by sex for all ages.

**Table 4:** Top 20 disease and injury causes with the highest DALYs by sex, all ages, Iran 2003

**Males**	**DALY**	**Females**	**DALY**
Road traffic injuries	1070969	Ischemic heart disease	437709

Natural disasters ^(1)^	548299	Major depressive disorder	420475

Opioid use ^(2)^	510291	Natural disasters ^(1)^	419781

Ischemic heart diseases	433627	Road traffic injuries	234745

Falls	274038	Cerebrovascular disease	206869

Exposure to mechanical forces	202494	Low back pain	199896

Major depressive disorder	181101	Knee osteoarthritis	196343

Cerebrovascular disease	146770	Anemias	165411

Bipolar disorders	139501	Bipolar disorders	128402

COPD ^(3)^	128230	Burns	105194

Burns	114677	COPD ^(3)^	104381

Low back pain	107876	Panic disorder	100129

Anemias	104169	Diabetes Mellitus	99670

Calculus of kidney and ureter	95688	Falls	90206

Knee osteoarthritis	94962	Obsessive-compulsive disorder	88449

Premature birth with normal weight	93740	Schizophrenia and schizo-affective disorders	82582

Perinatal respiratory and cardiovascular disorders ^(4)^	84779	Menopause (hot flushes & atrophic vaginitis)	76314

Intentional self-harm	84424	Acne vulgaris	75689

Premature birth with low weight	78850	Upper respiratory infections and otitis media	74009

Assault	76797	Premature birth with normal weight	73102

(1) Natural disasters (exposure to forces of nature) including the Bam earthquake of 26 December 2003; (2) Mental and behavioral disorders due to use of opioids or opioids with multiple drugs; (3) COPD: Chronic Obstructive Pulmonary Disease; (4) Respiratory and cardiovascular disorders specific to the perinatal period

The burden of musculoskeletal, genitourinary, endocrine, nutritional and metabolic diseases, and mental and behavioral disorders was higher in females, whereas males had more burden of intentional and unintentional injuries, neoplasms, infectious and parasitic diseases, and perinatal conditions (in descending order of DALY rates differences). These differences are important with respect to primary and secondary prevention approaches. The burden of disease from road traffic injuries in males was about five times higher than that in females, from mental disorders due to opioid use – about ten times higher than that in females, and from falls – about three times higher than that in women. The burden of major depressive disorder, low back pain, and knee osteoarthritis in females was about two times higher than the respective burden levels in males. Naturally, the burden from hemorrhagia due to fibroids and other causes, and maternal conditions affected exclusively females. In general, it seems that the burden due to acute and relatively early consequences of occupational and social problems fell more on males, whereas burden due to chronic and late consequences of occupational, social, and bodily problems affected mainly females.

There was an extra burden of disability in females between the ages of 15–69, as compared with males, especially in the 40–65 year range. On the other hand, an extra burden of death affected males between the ages of 5 and 69, in comparison with females, especially in the 20–64 age groups (see figure 3). The DALY rates values against age resembled a check mark, with the lowest values at age 5–14, returning to at-birth values at age 60–69, and the highest values at the highest age of 80 or older. Females had lower DALY rates than males in all age groups, except in the 45–64 one.

### (C) Burden by disease type and age

**Figure 4:**
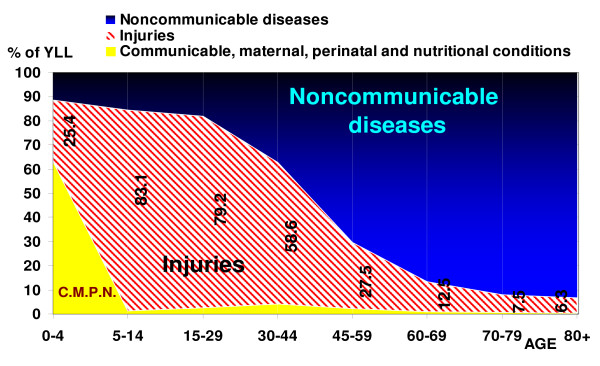
**Share of YLL by three GBD disease groups and age, Iran 2003**. Figures in black indicate the percent of YLL due to injuries to total YLL in each age group.

**Figure 5:**
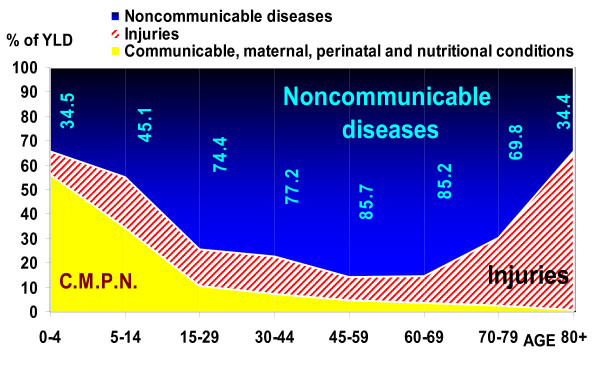
**Share of YLD by three GBD disease groups and age, Iran 2003**. Figures in blue indicate the percent of YLD due to noncommunicable diseases to total YLD in each age group.

**Figure 6:**
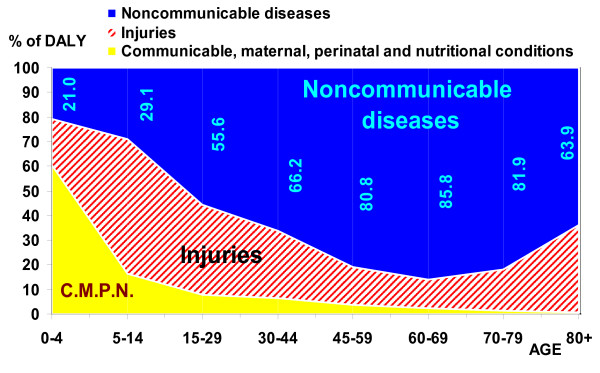
**Share of DALYs by three GBD disease groups, Iran 2003**. Figures in blue indicate the percent of DALYs due to noncommunicable diseases to total DALYs in each age group.

Injuries (group III of GBD) caused the highest proportion of mortality burden in the 5–44 year age group, and noncommunicable diseases were the leading cause of this burden in the population older than 44 years, for both sexes. Noncommunicable diseases were the leading cause of disability burden in the 15–69 year age group, particularly mental disorders such as opioid use and major depressive disorder; cardiovascular diseases such as ischemic heart disease and cerebrovascular disease; musculoskeletal diseases such as knee arthritis and low back pain; genitourinary diseases in women, such as fibroids and consequences of menopause, and other diseases such as anemia, skin disease, and diabetes mellitus. Injuries caused the highest proportion of YLD in the population older than 80 years, most notably due to falls, and often aggravated by osteoporosis. Perinatal conditions caused the most disability burden (YLD) in the 0–4 year age group. Noncommunicable diseases were the leading cause of the total burden of mortality and disability (DALYs) in the population older than 15. The Injuries group was responsible for the highest total burden in the 5–14 year age group, and perinatal conditions, malnutrition and its consequences such as iron deficiency anemia caused the highest total burden in the 0–4 year age group. The burden is measured by DALY rates per 100,000, and proportions of the absolute number of years lost among age groups are not the same (see figures 4, 5 and 6). Appendix table S2 shows the top eleven disease and injury causes with the highest DALY rates by age groups in both sexes [see Additional file 2].

### (D) Burden of disease estimation for six provinces of Iran

**Figure 7:**
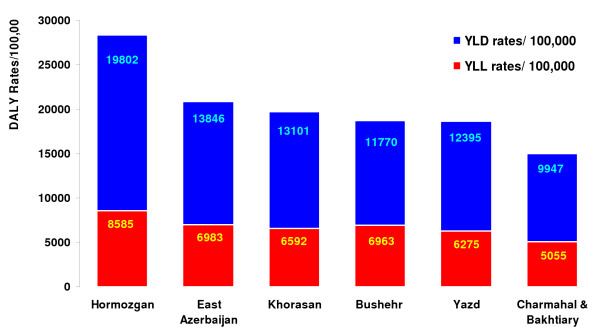
**Total YLD and YLL rates per 100,000 in six provinces of Iran in 2003**.

YLL and YLD for six provinces of Iran were calculated for different age groups and both sexes; these estimates were based on epidemiological estimates at the province level in many cases. Comparison of the findings with national results showed that intentional and unintentional injuries had the highest DALY rates in all six provinces, which resembled the picture at the national level. In a similar way, mental and behavioral disorders had the second highest DALY rates in the selected six provinces. The differences among the provinces as well as with the national average burden started from the disease with the third highest DALY rates, based on different socioeconomic determinants and epidemiological settings (see figure 7). Table 5 shows the order of disease groups by DALYs in these six provinces.

**Table 5:** Top 11 disease groups with the highest burden (DALY rates/100,000) in six provinces, all ages and both sexes, Iran 2003

	**Provinces and disease groups**						
	
**Rank**	**National level**	**East Azerbaijan**	**Bushehr**	**Charmahal Bakhtiary**	**Hormozgan**	**Khorasan**	**Yazd**
1	Injuries	Injuries	Injuries	Injuries	Injuries	Injuries	Injuries

2	Mental ^(1)^	Mental ^(1)^	Mental ^(1)^	Mental ^(1)^	Mental ^(1)^	Mental ^(1)^	Mental ^(1)^

3	Circulatory	Circulatory	Circulatory	Circulatory	Infectious	Circulatory	Circulatory

4	Perinatal	Musc.-skel. ^(2)^	Congenital ^(3)^	Musc.-skel. ^(2)^	Circulatory	Musc.-skel. ^(2)^	Musc.-skel. ^(2)^

5	Musc.-skel. ^(2)^	Respiratory	Perinatal	Perinatal	Perinatal	Perinatal	Respiratory

6	Genitourin. ^(4)^	Genitourin. ^(4)^	Musc.-skel. ^(2)^	Genitourin. ^(4)^	Blood system^(5)^	Genitourin. ^(4)^	Endocrine ^(6)^

7	Digestive	Neoplasms	Genitourin. ^(4)^	Digestive	Respiratory	Digestive	Genitourin. ^(4)^

8	Respiratory	Digestive	Digestive	Respiratory	Musc.-skel. ^(2)^	Respiratory	Perinatal

9	Endocrine ^(6)^	Perinatal	Endocrine ^(6)^	Skin ^(7)^	Digestive	Infectious	Neoplasms

10	Neoplasms	Endocrine ^(6)^	Neoplasms	Neoplasms	Congenital ^(3)^	Neoplasms	Digestive

11	Nervous ^(8)^	Infectious	Nervous ^(8)^	Endocrine ^(6)^	Genitourin. ^(4)^	Endocrine ^(6)^	Nervous ^(8)^

(1) Mental and behavioral disorders; (2) Musculoskeletal diseases; (3) Congenital malformations and chromosomal abnormalities; (4) Genitourinary diseases; (5) Blood and blood forming organs diseases; (6) Endocrine, nutritional and metabolic diseases; (7) Skin and subcutaneous tissues diseases; (8) Nervous system diseases

### (E) Regional comparisons

**Figure 8:**
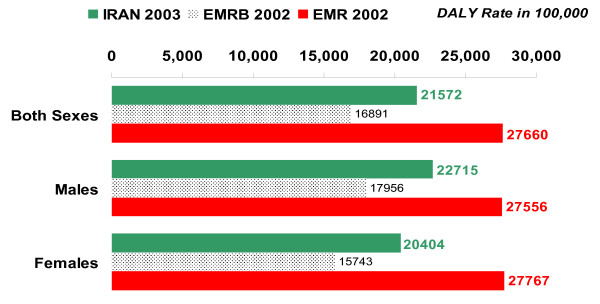
**Total DALY rates per 100,000 by sex, all ages, Iran 2003, EMR Sub-region B (EMRB) 2002, and Eastern Mediterranean Region (EMR) 2002**.

**Figure 9:**
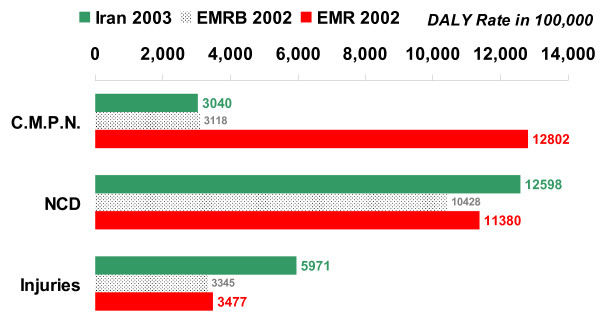
**DALY rates per 100,000, by three disease groups of GBD, all ages and both sexes, Iran 2003, EMR Sub-region B (EMRB) 2002, and Eastern Mediterranean Region (EMR) 2002**. C.M.P.N.: Communicable, Maternal, Perinatal, and Nutritional conditions; NCD: Noncommunicable Diseases.

**Figure 10:**
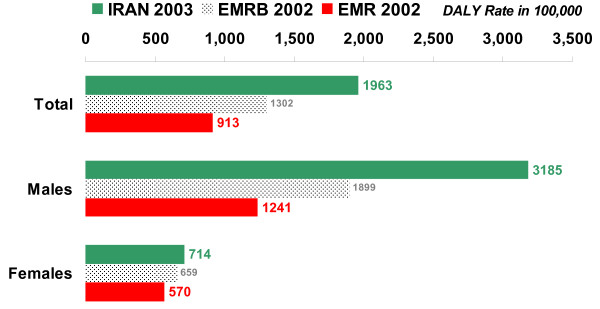
**DALY rates per 100,000 from road traffic injuries by sex, all ages, Iran 2003, EMR Sub-region B (EMRB) 2002, and Eastern Mediterranean Region (EMR) 2002**.

The total burden of disease (DALY rate per 100,000) in Iran in 2003 was higher than the WHO estimate of burden in EMR-B in 2002 and lower than that in EMR in 2002 (see figure 8). The burden of communicable, maternal, perinatal, and nutritional conditions (as a group) was lower in Iran than in EMR and EMR-B, and the burden of injuries was higher in Iran than in these regions (see figure 9). Within the former group, communicable diseases and maternal conditions had much lower burden in Iran than in both EMR-B and EMR. The burden of noncommunicable diseases in Iran was lower than that in EMR but higher than that in EMR-B. The burden due to perinatal and nutritional conditions in Iran was higher than that in EMR-B and lower than the burden due to these causes in EMR. While the burden of cardiovascular disease was relatively similar in Iran, EMR and EMR-B, the burden of neuropsychiatric disorders was higher in Iran, mainly due to the burden of major depressive disorder and mental disorders due to opioid use. Excess burden of major depressive disorder was observed mainly among the middle-aged females and extra burden of mental disorders due to opioid use – among middle-aged males. The burden of both unintentional and intentional injuries was nearly two times higher in Iran than in EMR-B and EMR, mainly due to the higher burden of injuries in Iranian males. The major part of this higher burden was observed in young and middle-aged Iranian males as victims of road traffic injuries (see figure 10).

## Discussion

### (A) Health transition

The most prominent finding of the first national burden of disease study in Iran for 2003, as indicated by the mix and levels (rates) of YLD, YLL, and DALYs caused by three groups of noncommunicable diseases, injuries, and "communicable, maternal, perinatal, and nutritional conditions," was an advanced health transition profile from communicable, maternal, and perinatal conditions and nutritional deficiencies, through injuries, to the dominance of noncommunicable diseases in the overall picture of disease burden. Assessment of mortality and morbidity rates had demonstrated the dominance of noncommunicable, maternal, perinatal, and nutritional conditions in Iran in recent four decades, as well as the start of the health transition in the past few years [31-33]. While this was the prominent overall national picture, results at the province level showed the variability of health transition advancement among provinces, which can be due to differences in the relative socioeconomic development levels of the studied provinces. Whereas the overall dominance of injuries and noncommunicable diseases was observed for the top three most burdensome disease groups in all six provinces, the Hormozgan province was an exception to this; there infectious and parasitic diseases (including malaria) caused the third highest burden. Maternity-related disease burden ranked last, due to the advances in the reduction of maternal mortality, but the burden of perinatal conditions was much higher, mainly due to the difficulties in reducing neonatal disease burden.

### (B) Implications for health policies and programs

The mix of causes for YLL and YLD shows reducing the burden of which disease groups can extend the total life expectancy (relatively more than increasing the healthy life expectancy), and proper management of which other disease groups is expected to have a larger effect on reducing disability and increasing the healthy life expectancy (as compared with the resultant increase in total life span). Hence, by decreasing the perinatal mortality and the frequency and intensity of road traffic injuries, the total life expectancy can be improved (with relatively less increase in healthy life expectancy), since these disease and injury causes have very high YLL to YLD ratios. On the other hand, through reducing the incidence of and increasing the quality of healthcare for noncommunicable diseases such as ischemic heart disease, major depressive disorder, and mental disorders due to opioid use, loss of healthy years of life can be prevented, and the relative gain in healthy life expectancy should be higher than the relative increase in total life expectancy. Across the age- and sex-specific subgroups, as well as in different geographical regions, the priority areas for intervention differ, a matter of crucial importance for customizing the priority diseases' control projects over person and place variables. The use of results in priority setting is discussed below.

### (C) Comparison of Iran's National Burden of Disease study with international estimates and their differences

We compared our study results with WHO estimates for subregion B of Eastern Mediterranean Region (EMR-B) for year 2002 since the latter set of burden estimates had the nearest reference year of calculations to our own reference year and Iran is located in this subregion of EMR. Details of comparisons for DALY, YLL, and YLD at cause groups and specific cause levels are provided in Additional File 3. Some differences exist between Iran's NBD and WHO estimates for EMR-B, the most important ones of which are described below along with the most possible reasons for the differences.

#### C1: YLL

Estimate of all-cause YLL rate in 100000 population in our study is higher than WHO's estimate for EMR-B. The main reason for this difference is inclusion of about 30000 deaths from Bam earthquake of December 2003 in our study. If the YLD due to Bam earthquake not included, our all-cause YLL would in fact be even slightly lower than estimate for EMR-B by WHO.

#### C2: YLD

YLD rate estimate in our study is higher than that by WHO for EMR-B for all-cause and all-ages and for some of the causes and over most age groups. These differences should be due to the following facts.

**a**. We omitted 18 causes form the GBD cause list (9 causes in cluster I, 8 causes in cluster II, and one cause in cluster III), which are not existent in Iran or are in elimination and eradication phase, or there was not any information or study available about their prevalence estimates in Iran. On the other hand, we added 92 causes to the GBD cause list, as described above under 'Choosing the diseases' in Methods section. Therefore, strict comparisons of YLL and YLD for and at all-cause, cluster, or disease group levels would not reach rational results. Moreover, addition of the above-mentioned causes changes the age and sex distribution of YLD rates within clusters, since the added causes are mainly of high YLD and more incident among women. Table S8 in Additional File 3 shows these diseases.

**b**. For all causes present in GBD cause list, we used the disability weights from GBD study directly without adjusting for differences in case-fatality in Iran. For causes not present in GBD cause list, disability weights were taken from Dutch Disability Weights Group [11] or reproduced as described under 'Disability weights' in Methods section. Similarly, the same disease or disability durations were used from GBD study method, unless for the causes whose GBD duration values were inconsistent with the actual duration times in Iran. For such causes, duration was estimated using Iranian clinical specialists and disease epidemiology experts' views and studies. The important point is the differences in proportion of patients who had access to treatment and utilized the services, and in effectiveness of such treatments in terms of reducing the disability duration, that existed for some causes between the actual circumstances in Iran and those circumstances leading to GBD disability duration estimates. Where such differences became evident according to available evidence from studies and expert views in Iran, disease duration estimates based on local evidence were used instead of GBD duration estimates. Prominent examples include effective treatment coverage for cancers, ischemic heart diseases, and injuries, especially falls with femoral neck fractures among the elderly. For duration estimation of cancers, we used survival data from Iran and for ischemic heart diseases, and injuries we forced the estimated durations into DISMOD.

**c**. Part of the differences in YLD between our results and WHO estimates are due to differences in age distribution of some disease frequencies. Many studies existed in Iran that revealed the age patterns of disease frequencies in Iran which were different from those used for WHO estimates and these studies' results were used in our estimations. Examples include iron deficiency anemia, drug use disorders, bipolar disorders, and road traffic injuries.

**d**. There are clear differences in YLD rate estimates for some of the causes in GBD list between the present study and WHO estimates, whose possible reasons are described below.

##### - Drug use disorders

In Iran, these causes mainly included addiction to opium, heroin, opium-derivative containing medicines, and tranquilizer medicines. Prevalence rate estimates for these disorders were taken from Epidemiologic Study of Drug Abuse in Iran for year 2001 [24]. Incidence estimates for males and males were 4.7 and 0.75 in 1000 respectively (with different age distributions). Cause-specific mortality rate estimates were 0.07 and 0.003 in 1000 for males and females respectively. Proportion of treated patients was less than 0.2 in 2003 and disease duration surpassed 3 years. Disability weight was the same as that in GBD indeed. The above-mentioned study showed that based on DSM-IV definitions, 1.5 million opium addicts, 120 thousand heroin addicts, and 5 million opium abusers (irregular and non-dependent abusers) existed in Iran in 2001 [24]. Only the two former figures (opium and heroin addicts) were included for burden estimation in this study. The observed difference in YLD estimates for drug abuse disorders between our estimates and WHO estimates for EMR-B is only due to our higher incidence rate and difference in its age distribution and lengthier disease duration.

##### - Road traffic injuries (RTI)

WHO's GBD study for year 2002 showed that Iran had the highest mortality rate from road traffic accidents and ranked third among all countries for its DALYs. Therefore, Iran's estimates for RTI burden are naturally much higher than those for the EMR-B subregion.

##### - Falls

Our study estimates for YLD from falls among the elderly were much higher than WHO estimates for EMR-B, a difference which is principally due to higher incidence of falls with femoral neck fracture in Iranian elder population, according to a study on epidemiology of injuries due to external causes in Iran [8], limited access to appropriate treatment for femoral neck fracture, and the resultant elongated duration of disability ensuing from the fracture. Therefore, the higher incidence rates and disability duration as compared with WHO study are the reasons for the observed differences.

##### - Low back pain

This cause included cervical arthrosis and pain, high back pain, and lumbar pain with or without sciatica in our study. Prevalence rate estimates were taken from COPCORD study in Iran [26]. For cervical and lumbar pains without radiation to extremities, mean duration was 14 days with 85% remission and 42 days with 100% remission. For lumbar pain with sciatica, mean duration was 28 days with 85% remission and 42 days with 100% remission. Differences between our study and WHO's are due to different disease definitions and age and sex specific incidence rates.

##### - Osteoarthritis

Based on available pertinent studies in Iran, we estimated the YLD for unilateral and bilateral knee osteoarthritis, and other types of unilateral and bilateral osteoarthritis (pelvic, etc.) were not included. Mortality and definitive remission were not considered for knee osteoarthritis, but only flare-up and remission periods were taken into calculations. Disability weight for bilateral knee osteoarthritis does not exist in GBD and hence it was reproduced for our study. The observed differences between our estimates and those by WHO are due to lack of access to effective treatment, differences in disability durations, and addition of bilateral form of disease into burden estimation in Iran.

##### - Ischemic heart disease

Three distinct forms were included in our study based on the available information sources. *(1) *Acute myocardial infarction and imminent death before access to hospital: This subgroup of patients with acute myocardial infarction died without access to hospital, before reaching to hospital, and before receiving effective life-saving treatments within hospitals in Iran: 9% of males and 17% of females in 30–44 year age group, 8% of males and 16% of females in 45–59 year, 2% of males and 3% of females in 60–69 year, 13% of males and 20 of females in 70–79 year, and 43% of males and 58% of females in 80 year and above [27,28]. YLL but not YLD was estimated for this subgroup, since mean disease duration from infarction to death was estimated as only 6 hours. *(2) *Angina pectoris: No mortality was regarded for this group while they had angina only. Disease duration before exiting this disease and entering other diseases or remission was different across age groups. Mean duration for all ages and both sexes was 3.5 years. Disability weight was the same as GBD's. *(3) *Acute myocardial infarction patients who found access to hospital admission and treatment, finally remitted and were discharged from hospital after 24 days (80%), died after 3 months (15%), or entered heart failure (5%). The latter group died after 3 months (5%) or entered a compensated heart failure status (95%), with a mean duration of 6 years [27,28]. Disability weights for myocardial infarction and heart failure were the same as GBD weights. Differences between our estimates and WHO's are due to differences in incidence rates, their age patterns, and disability durations based on different mix of access to effective treatment services.

##### - Panic disorders

Prevalence and incidence rates were adopted from a large national survey for psychiatric disorders [23]. Difference in age pattern of incidence is the main cause of difference of YLD due to panic disorder between our study and WHO estimates, as shown in figure S12 of the Additional File 3. Disability weight was the same as GBD's and no mortality was included.

**e**. From group of diseases included in Iran's study but not in WHO estimates, two examples with clinically observable YLD ample.

##### - Acne vulgaris

Incidence rate estimate for both sexes was 11% in 15–29 year age group and 2% in 30–45 year. Duration was 2.5 and 1.7 years for these two age groups respectively, without any mortality. Disability weight was equal with that for eczema from Dutch Disability Weights Group [11], or 0.056.

##### - Menopause

For this period of female life that is accompanied with pathophysiologic consequences in most cases, two distinct conditions were included in YLD estimation separately and their sum of YLD accounted as YLD due to menopause. These two conditions were menopausal climacteric states (symptoms such as flushing, sleeplessness, headache, lack of concentration) with ICD code N59.1 and postmenopausal atrophic vaginitis with ICD code N59.2. Incidence rates were estimated as 7.8% and 2.9% respectively for N59.1 and N59.2 among the females of 45–59 year age. Disability duration was estimated as equal to two years for the two conditions consecutively (not as separate episodes) based on clinical specialists' opinion. Disability weights were estimated as 0.1 for N95.1 and 0.03 for N95.2 based on simulation with similar conditions by the clinicians' opinion.

### (D) Study limitations and innovations

Epidemiological estimations of disease status transition rates used as inputs for disease modeling are generally based on more extensive and accurate information in developed countries, as compared with those in developing countries, due to the less developed disease surveillance, death registry, and biomedical and health research policies and practices in the latter group. Nevertheless, we reviewed all the available published and unpublished information needed for epidemiological disease modeling in Iran. Some information came from studies with subnational coverage, and assumptions were made about their generalizability to the national population. We also used a modeling approach for estimation of the total population and its subgroups, based on extrapolations of the most recent intercensal period. It is not expected that rankings of the disease groups (by GBD groups or major disease groups) or even ranks of the most burdensome detailed causes within each disease group are very sensitive to these assumptions. Lack of full coverage for death data of the 5 out of 28 provinces in 2003 did not affect much the estimations of mortality and YLL rates and did not affect the ranking of the cause groups by YLL or DALYs, because the values of these rates for the 23 provinces were not very different from those for the 28 provinces, the latter obtained later as described below. Mortality and YLL rates for the 23 provinces in 2003 were 438 and 6,412 per 100,000 respectively (without taking into account the Bam earthquake). Mortality data for four out of the five provinces that were not fully covered by the death registration system in 2003 was obtained in 2004. Mortality and YLL rates for all provinces of Iran, except the Tehran province, were 451 and 6,661 per 100,000 respectively in 2004, and 434 and 6,269 in 2005 [34,35]. Besides these six mentioned values for mortality and YLL rates, all other rates are estimated and presented taking the Bam earthquake into account. We used disability durations for disease and injuries mainly from GBD study method, except for cancers, ischemic heart diseases, and injuries.

Adaptations of the methods recommended by WHO for the National Burden of Disease studies were made, some of which could be considered innovative and value-adding. The burden of disease was estimated at the subnational level, although only in six provinces.

## Conclusion

The health and disease profile in Iran has made the transition from the dominance of communicable diseases to that of noncommunicable diseases and injuries. NBD results are to be used in health program planning, research, and resource generation policies and practices.

### (A) Use of the current study's results

Burden of disease results provide a crucial part of biomedical information needed for evidence-based health policymaking. This information along with economic analyses of the cost-effectiveness of interventions forms a strong platform for the advancement of evidence use in health policy and management. In essence, the diseases, injuries, and risk factors causing the highest burden should be assessed with respect to the current evidence, its accuracy and national applicability, on the effectiveness and cost-effectiveness of available prevention and control interventions, and the availability of organizational, human, financial, and technological resources. The NBD results can provide strategic directions for population health research, resource generation and expansion, health programs evaluation, health system development, and future forecasting. This opportunity can be viewed and used as a substantial advancement in evidence-based health policymaking and program planning.

### (B) Future rounds of Iran's NBD study

The NBD study is to be conducted every five years, in order to coincide with the five-year cycle of the Comprehensive Social and Economic Development Master Plan of I. R. Iran. The second IRNBD study is being launched to make 2005 estimates.

## Abbreviations

BOD: Burden of Disease; DALY: Disability-Adjusted Life Year; DHD: District Health Department; DHS: Demographic and Health Survey; DISMOD: Disease Modeling (software); EMR: Eastern Mediterranean Region; EMR-B: Eastern Mediterranean Region, sub-region B; EMRO: Eastern Mediterranean Region Office (of WHO); GBD: Global Burden of Disease; HALE: Health-Adjusted Life Expectancy; HSPA: Health System Performance Assessment; IRNBD: (The first) Iranian National Burden of Disease Study; MOHME: Ministry of Health and Medical Education (Iran); NBD: National Burden of Disease; SMPH: Summary Measures of Population Health; WHO: World Health Organization; YLD: Years Lived with Disability; YLL: Years of Life Lost due to premature mortality.

## Competing interests

The authors declare that they have no competing interests.

## Authors' contributions

MN conceived of and led the study, adopted the methodology, and performed the statistical analysis of all National Burden of Disease (NBD) study components. FA contributed to the study's framework and methodology, and developed the specific template for modeling the cancer epidemiology. MML developed the specific template for modeling the injury epidemiology and conducted most of the DISMOD calculations. MN designed and managed the NBD mortality and disability data development and processing. MN, MML, NJ, SV, NMH, HK, and FP contributed to the NBD data development and processing. FP and MN drafted the manuscript and all authors read and approved the final manuscript.

## Supplementary Material

Additional file 1 **Mortality rates in 100'000 population by age, sex, and cause; Iran, 2003**. Detailed tabulations of deaths, YLL, YLD, and DALYs for the 213 causes included in Iranian National Burden of Disease study, classified by GBD and ICD groups, age, and gender. Click here for file

Additional file 2 **Disability weights from the GBD and Dutch studies and weights developed by the Iranian national burden of disease study team**. Disability weights for all causes in Iranian National Burden of Disease study. The top eleven disease and injury causes with the highest DALY rates by age groups in both sexes. Click here for file

Additional file 3 **Detailed comparison of Iranian National Burden of Disease study results with WHO's estimations for EMR-B 2002**. We compared Iran's NBD study results with WHO estimates for 2002. Here we describe the differences and conclude about the reasons for their existence. Click here for file
