# Supplementary material for: The burden of disease and injury in Iran 2003
Source: Popul Health Metr. 2009 Jun 15;7:9. doi: 10.1186/1478-7954-7-9 (PMC2711041; doi:10.1186/1478-7954-7-9)
Supplement: Additional file 2 — Disability weights from the GBD and Dutch studies and weights developed by the Iranian national burden of disease study team. Disability weights for all causes in Iranian National Burden of Disease study. The top eleven disease and injury causes with the highest DALY rates by age groups in both sexes. [file 1478-7954-7-9-S2.doc]

Appendix table S1. Disability weights from the GBD and Dutch studies * and weights developed by the Iranian national burden of disease study team

* Reproduced from Mathers C, Vos T, Stevenson C. The burden of disease and injury in Australia. Canberra, Australian Institute of Health and Welfare, 1999. http://www.aihw.gov.au/publications/phe/bdia/bdia-c00.pdf

| **Disease category, subcategory, or sequelae** | **Disability weight** | **Comments** |
| --- | --- | --- |
| **I. Communicable diseases, maternal and neonatal** **conditions** |  |  |
| **A. Infectious & parasitic diseases** |  |  |
| 1. Tuberculosis |  |  |
| Pulmonary tuberculosis | 0.295 | GBD weight |
| Extra-pulmonary tuberculosis | 0.300 | GBD weight |
| 2. Sexually transmitted diseases (not HIV/AIDS) |  |  |
| a. Syphilis |  |  |
| Primary syphilis | 0.148 | GBD weight |
| Secondary syphilis | 0.048 | GBD weight |
| Tertiary syphilis (cardiovascular) | 0.196 | GBD weight |
| Tertiary syphilis (gummas) | 0.102 | GBD weight |
| Tertiary syphilis (neurologic) | 0.283 | GBD weight |
| Syphilis (congenital) | 0.315 | GBD weight |
| b. Chlamydia |  |  |
| Conjunctivitis | 0.180 | GBD weight |
| Urethritis | 0.067 | GBD weight |
| Cervicitis | 0.049 | GBD weight |
| Pelvic inflammatory disease | 0.420 | GBD weight |
| Ectopic pregnancy | 0.549 | GBD weight |
| Chronic pelvic pain | 0.122 | GBD weight |
| Infertility | 0.180 | GBD weight |
| Tubo-ovarian abscess | 0.549 | GBD weight |
| c. Gonorrhea |  |  |
| Urethritis | 0.067 | GBD weight |
| Cervicitis | 0.049 | GBD weight |
| Pelvic inflammatory disease | 0.420 | GBD weight |
| Ectopic pregnancy | 0.549 | GBD weight |
| Chronic pelvic pain | 0.122 | GBD weight |
| Infertility | 0.180 | GBD weight |
| Tubo-ovarian abscess | 0.549 | GBD weight |
| d. Other sexually transmitted disease |  |  |
| Pelvic inflammatory disease | 0.420 | GBD weight |
| Ectopic pregnancy | 0.549 | GBD weight |
| Chronic pelvic pain | 0.122 | GBD weight |
| Infertility | 0.180 | GBD weight |
| Tubo-ovarian abscess | 0.549 | GBD weight |
| 3. HIV/AIDS |  |  |
| Diagnosed asymptomatic HIV | 0.200 | Dutch weight |
| Symptomatic HIV | 0.310 | Dutch weight |
| AIDS | 0.560 | Dutch weight |
| AIDS—terminal phase | 0.950 | Dutch weight |
| 4. Diarrheal diseases and gastroenteritis |  |  |
| Uncomplicated episode | 0.093 | GBD age-specific weights. Average shown here |
| Complicated episode | 0.420 | Dutch weight for complicated episode (50%) plus GBD weight for uncomplicated episode (50%) |
| 5. Childhood immunisable diseases |  |  |
| a. Diphtheria |  |  |
| Cases | 0.230 | GBD weight |
| Neurological complications | 0.078 | GBD weight |
| Myocarditis | 0.323 | GBD weight |
| b. Whooping cough |  |  |
| Pertussis episode | 0.178 | GBD weight |
| Mental retardation (treated) | 0.420 | GBD weight (0.394 0–4 years, 0.420 5–14 years) |
| Mental retardation (untreated) | 0.483 | GBD weight (0.469 0–4 years, 0.483 5–14 years) |
| c. Tetanus |  |  |
| Cases | 0.612 | GBD weight |
| d. Poliomyelitis |  |  |
| Poliomyelitis | 0.369 | GBD weight |
| e. Measles |  |  |
| Episodes | 0.152 | GBD weight |
| Measles encephalitis | 0.338 | GBD weight for neurological sequelae of encephalitis |
| Sub-acute sclerosing panencephalitis | 0.930 | Dutch weight for end-stage disease |
| f. Rubella |  |  |
| Episodes | 0.152 | GBD weight for measles episode |
| Congenital cataract | 0.430 | Dutch weight for severe vision loss |
| Congenital heart disease | 0.350 | Dutch weight for heart failure |
| Congenital deafness | 0.230 | Dutch weight |
| g. Hemophilus influenzae type b (Hib) |  |  |
| Epiglottitis | 0.152 | GBD weight for hemophilus influenzae episode |
| Meningitis | 0.430 | Average of weights for meningitis manifestations |
| Septicemia | 0.350 | GBD weight |
| Pneumonia | 0.230 | Estimated using EQ5D + regression model |
| 6. Meningitis |  |  |
| Acute episode | 0.913 | Estimated using EQ-5D+ regression model |
| After effects up to 6 months | 0.226 | Estimated using EQ-5D+ regression model |
| VP shunt | 0.170 | Dutch weight for motor deficit |
| Hearing loss | 0.234 | Average of Dutch weights for mild, moderate, and severe loss |
| Seizure disorder | 0.110 | Dutch weight |
| Less severe developmental problems | 0.100 | Average of Dutch weights for developmental problems |
| Mental retardation | 0.250 | Dutch weight |
| Motor deficit + mental retardation | 0.760 | Dutch weight |
| Less severe developmental problems | 0.100 | Based on Dutch weights for developmental problems |
| Scarring/deformity | 0.133 | Based on GBD amputation weights |
| 7. Septicemia |  |  |
| Cases | 0.613 | GBD age-specific weights (average shown here) |
| 8. Arbovirus infection (incl. Ross River fever) |  |  |
| a. Ross River virus Infection |  |  |
| Acute phase | 0.258 | Dutch weight for moderate rheumatoid arthritis |
| Chronic phase | 0.140 | Dutch weight for mild rheumatoid arthritis |
| b. Barmah Forest virus |  |  |
| Acute phase | 0.258 | Dutch weight for moderate rheumatoid arthritis |
| Chronic phase | 0.140 | Dutch weight for mild rheumatoid arthritis |
| c. Other arbovirus infection |  |  |
| Australian encephalitis | 0.613 | GBD weight for Japanese encephalitis |
| Japanese encephalitis | 0.613 | GBD weight |
| Kunjun | 0.613 | GBD weight for Japanese encephalitis |
| Cognitive impairment | 0.451 | GBD weight |
| Neurological sequelae | 0.334 | GBD weight |
| d. Dengue fever |  |  |
| Dengue hemorrhagic fever | 0.172 | GBD age-specific weights (average shown here) |
| 9. Hepatitis |  |  |
| a. Hepatitis A |  |  |
| Uncomplicated episode | 0.093 | GBD age-specific weights. Average shown here |
| Complicated episode | 0.420 | Dutch weight for complicated episode (50%) plus GBD weight for uncomplicated episode (50%) |
| Prolonged or relapsing episode | 0.140 | Dutch weight for mild depression. |
| b. Hepatitis B |  |  |
| Cases | 0.000 | Asymptomatic cases only |
| Acute symptomatic episode | 0.210 | Dutch weight |
| Chronic symptomatic carrier | 0.360 | Dutch weight |
| Compensated liver cirrhosis | 0.310 | Dutch weight |
| Decompensated liver cirrhosis | 0.840 | Dutch weight |
| Hepato-cellular cancer | — | See sequelae and weights for F5. Liver cancer |
| c. Hepatitis C |  |  |
| Cases | 0.000 | Asymptomatic cases only |
| Acute symptomatic episode | 0.210 | Dutch weight for Hepatitis B |
| Chronic symptomatic carrier | 0.360 | Dutch weight for Hepatitis B |
| Compensated liver cirrhosis | 0.310 | Dutch weight |
| Decompensated liver cirrhosis | 0.840 | Dutch weight |
| Hepato-cellular cancer | — | See sequelae and weights for F5. Liver cancer |
| 10. Malaria |  |  |
| Episodes | 0.175 | GBD age-specific weights (average shown here) |
| Neurological sequelae (treated) | 0.436 | GBD weight for 0–4 years |
| Anemia | 0.012 | GBD age-specific weights (average shown here) |
| 11. Trachoma |  |  |
| Moderate vision loss | 0.170 | Dutch weight |
| Severe vision loss | 0.430 | Dutch weight |
| **B. Acute respiratory infections** |  |  |
| 1. Lower respiratory tract infections |  |  |
| Influenza episode | 0.047 | Estimated using EQ-5D + regression model |
| Acute bronchitis episode | 0.132 | Estimated using EQ-5D + regression model |
| Pneumonia episode | 0.373 | Estimated using EQ-5D + regression model |
| 2. Upper respiratory tract infections |  |  |
| Acute nasopharyngitis | 0.014 | Estimated using EQ-5D + regression model |
| Acute sinusitis | 0.061 | Estimated using EQ-5D + regression model |
| Pharyngitis/tonsillitis | 0.061 | Estimated using EQ-5D + regression model |
| 3. Otitis media |  |  |
| Acute episodes | 0.090 | Dutch weight for 1 day severe pain plus 4 days moderate pain |
| Chronic otitis media | 0.110 | Dutch weight for early acquired mild to moderate hearing loss |
| Deafness | 0.233 | Dutch weight for early acquired severe hearing loss |
| **C. Maternal conditions** |  |  |
| 1. Maternal hemorrhage |  |  |
| Cases | 0.011 | GBD weight for moderate anemia |
| Severe anemia | 0.093 | GBD weight |
| 2. Maternal sepsis |  |  |
| Episodes | 0.000 | GBD weight |
| Infertility | 0.180 | GBD weight |
| 3. Hypertension in pregnancy |  |  |
| Episodes | 0.117 | Estimated using EQ-5D+ regression model |
| Neurological sequelae | 0.388 | GBD weight |
| 4. Obstructed labor |  |  |
| Episodes | 0.349 | Estimated using EQ-5D+ regression model |
| 5. Abortion |  |  |
| Episodes spontaneous abortion | 0.000 | GBD weight |
| Episodes induced abortion | 0.000 | GBD weight |
| Infertility | 0.180 | GBD weight |
| **D. Neonatal causes** |  |  |
| 1. Birth trauma & asphyxia |  |  |
| Deafness | 0.230 | Dutch weight |
| Seizure | 0.110 | Dutch weight |
| Cerebral palsy without intellectual disability | 0.170 | Dutch weight |
| Mild intellectual disability | 0.290 | Dutch weight |
| Moderate intellectual disability | 0.430 | Dutch weight |
| Severe intellectual disability | 0.820 | Dutch weight |
| Profound intellectual disability | 0.760 | Dutch weight |
| 2. Low birth weight |  |  |
| Mild permanent disability | 0.110 | Dutch weight for mild to moderate early acquired hearing loss |
| Severe hearing loss | 0.370 | Dutch weight |
| Vision loss | 0.170 | Dutch weight for moderate vision loss |
| Epilepsy | 0.110 | Dutch weight |
| Cerebral palsy without intellectual disability | 0.170 | Dutch weight |
| Mild intellectual disability | 0.290 | Dutch weight |
| Moderate intellectual disability | 0.430 | Dutch weight |
| Severe intellectual disability | 0.820 | Dutch weight |
| Profound intellectual disability | 0.760 | Dutch weight |
| 3. Neonatal infections |  |  |
| Acute neonatal episode | 0.894 | Dutch weight for acute meningitis episode |
| Deafness | 0.370 | Dutch weight |
| Motor deficit | 0.170 | Dutch weight |
| Mild intellectual disability | 0.290 | Dutch weight |
| Moderate intellectual disability | 0.430 | Dutch weight |
| Severe intellectual disability | 0.820 | Dutch weight |
| Profound intellectual disability | 0.760 | Dutch weight |
| 4. Other neonatal causes |  |  |
| Mild intellectual disability | 0.290 | Dutch weight |
| Moderate intellectual disability | 0.430 | Dutch weight |
| Severe intellectual disability | 0.820 | Dutch weight |
| Profound intellectual disability | 0.760 | Dutch weight |
| Cerebral palsy without intellectual disability | 0.170 | Dutch weight for motor deficit |
| **E. Nutritional deficiencies** |  |  |
| 1. Protein-energy malnutrition |  |  |
| Stunting | 0.002 | GBD Weight |
| Wasting | 0.053 | GBD Weight |
| Developmental disability | 0.024 | GBD Weight |
| 2. Iron-deficiency anemia |  |  |
| Non-anemic iron deficiency | 0.005 | Estimated using EQ-5D+ regression model |
| Mild anemia | 0.005 | GBD weight |
| Moderate anemia | 0.011 | GBD weight |
| Severe anemia | 0.090 | GBD weight |
| Very severe anemia | 0.250 | GBD weight |
| Cognitive impairment | 0.024 | GBD weight |
| 3. Other nutritional deficiencies |  |  |
| Iodine deficiency goiter | 0.026 | GBD weight for Grade 2 Goiter |
| **II. Noncommunicable diseases** |  |  |
| **F. Malignant neoplasms** |  |  |
| 1. Mouth and oropharynx cancers |  |  |
| Diagnosis and primary therapy | 0.560 | Dutch weight for esophageal cancer |
| State after intentionally curative primary therapy | 0.37 | Dutch weight for esophageal cancer |
| In remission | 0.370 | Dutch weight for esophageal cancer |
| Disseminated cancer | 0.900 | Dutch weight for esophageal cancer |
| Terminal stage | 0.930 | Dutch weight for end-stage disease |
| 2. Esophagus cancer |  |  |
| Diagnosis and primary therapy | 0.560 | Dutch weight |
| State after intentionally curative primary therapy | 0.37 | Dutch weight |
| Irradically removed or disseminated carcinoma | 0.900 | Dutch weight |
| Preterminal and terminal stages | 0.930 | Dutch weight for end-stage disease |
| 3. Stomach cancer |  |  |
| Diagnosis and primary therapy | 0.530 | Dutch weight |
| State after intentionally curative primary therapy | 0.38 | Dutch weight |
| Irradically removed or disseminated carcinoma | 0.730 | Dutch weight |
| Preterminal and terminal stages | 0.930 | Dutch weight for end-stage disease |
| 4. Colorectal cancer |  |  |
| Diagnosis and primary therapy | 0.430 | Dutch weight |
| State after intentionally curative primary therapy | 0.20 | Dutch weight |
| In remission | 0.430 | Dutch weight |
| Irradically removed or disseminated carcinoma | 0.830 | Dutch weight |
| Terminal stage | 0.930 | Dutch weight for end-stage disease |
| 5. Liver cancer |  |  |
| Diagnosis and initial treatment | 0.430 | Dutch weight for colorectal cancer |
| State after initially curative primary therapy | 0.200 | Dutch weight for colorectal cancer |
| Clinically disease free | 0.200 | Dutch weight for colorectal cancer |
| Irradically removed/disseminated/preterminal | 0.830 | Dutch weight for colorectal cancer |
| Terminal phase | 0.930 | Dutch weight for end-stage disease |
| 6. Gall bladder cancer |  |  |
| Diagnosis and initial treatment | 0.430 | Dutch weight for colorectal cancer |
| State after initially curative primary therapy | 0.200 | Dutch weight for colorectal cancer |
| Clinically disease free | 0.200 | Dutch weight for colorectal cancer |
| Irradically removed/disseminated/preterminal | 0.830 | Dutch weight for colorectal cancer |
| Terminal phase | 0.930 | Dutch weight for end-stage disease |
| 7. Pancreas cancer |  |  |
| Diagnosis and initial treatment | 0.430 | Dutch weight for colorectal cancer |
| State after initially curative primary therapy | 0.200 | Dutch weight for colorectal cancer |
| Disseminated | 0.830 | Dutch weight for colorectal cancer |
| Terminal phase | 0.930 | Dutch weight for end-stage disease |
| 8. Lung cancer |  |  |
| Diagnosis and primary therapy for operable | 0.440 | Dutch weight |
| non-small cell cancer |  |  |
| Disease free after primary therapy for | 0.470 | Dutch weight |
| non small cell cancer |  |  |
| Diagnosis and primary therapy for non operable non-small cell cancer | 0.760 | Dutch weight |
| Disseminated non-small cancer | 0.910 | Dutch weight |
| Terminal stage non small cell cancer | 0.930 | Dutch weight for end-stage disease |
| Diagnosis and chemotherapy small cell cancer | 0.680 | Dutch weight |
| Disease free after primary therapy for | 0.470 | Dutch weight |
| small cell cancer |  |  |
| Small cell cancer in remission | 0.540 | Dutch weight |
| Relapse/terminal stage small cell cancer | 0.930 | Dutch weight for end-stage disease |
| 9. Bone and connective tissue cancers |  |  |
| Diagnosis and primary therapy | 0.350 | Provisional weight based on Dutch weights |
| State after intentionally curative primary therapy | 0.30 | Provisional weight based on Dutch weights |
| In remission | 0.300 | Provisional weight based on Dutch weights |
| Disseminated carcinoma | 0.750 | Provisional weight based on Dutch weights |
| Terminal stage | 0.930 | Dutch weight for end-stage disease |
| 10. Melanoma |  |  |
| Primary treatment, no evidence dissemination | 0.190 | Dutch weight |
| No evidence of dissemination after initial treatment | 0.190 | Dutch weight |
| Primary treatment, lymph node but no distant dissemination | 0.430 | Dutch weight |
| In remission | 0.190 | Dutch weight |
| Disseminated melanoma | 0.810 | Dutch weight |
| Terminal phase | 0.930 | Dutch weight for end-stage disease |
| 11. Non-melanoma skin cancers |  |  |
| Basal cell carcinoma | 0.050 | Dutch weight |
| Squamous cell carcinoma undisseminated | 0.070 | Dutch weight |
| Squamous cell carcinoma with dissemination | 0.400 | Dutch weight |
| Squamous cell carcinoma–local recurrence | 0.500 | Dutch weight |
| Terminal phase | 0.930 | Dutch weight for end-stage disease |
| 12. Breast cancer |  |  |
| Diagnostic, primary therapy, non-invasive tumor <2 cm | 0.260 | Dutch weight |
| Diagnostic, primary therapy, tumor 2–5 cm or lymph node dissemination | 0.690 | Dutch weight |
| Diagnostic, primary therapy, tumor >5 cm | 0.810 | Dutch weight |
| Disease free after initial treatment | 0.260 | Dutch weight |
| In remission | 0.260 | Dutch weight |
| Disseminated cancer | 0.790 | Dutch weight |
| Terminal phase | 0.930 | Dutch weight for end-stage disease |
| 13. Cervix cancer |  |  |
| Diagnosis and primary therapy | 0.430 | Provisional weight based on Dutch weights |
| State after intentionally curative primary therapy | 0.20 | Provisional weight based on Dutch weights |
| In remission | 0.200 | Provisional weight based on Dutch weights |
| Disseminated carcinoma | 0.750 | Provisional weight based on Dutch weights |
| Terminal stage | 0.930 | Dutch weight for end-stage disease |
| 14. Uterus cancer |  |  |
| Diagnosis and primary therapy | 0.430 | Provisional weight based on Dutch weights |
| State after intentionally curative primary therapy | 0.20 | Provisional weight based on Dutch weights |
| In remission | 0.200 | Provisional weight based on Dutch weights |
| Disseminated carcinoma | 0.750 | Provisional weight based on Dutch weights |
| Terminal stage | 0.930 | Dutch weight for end-stage disease |
| 15. Ovary cancer |  |  |
| Diagnosis and primary therapy | 0.430 | Provisional weight based on Dutch weights |
| State after intentionally curative primary therapy | 0.20 | Provisional weight based on Dutch weights |
| In remission | 0.200 | Provisional weight based on Dutch weights |
| Disseminated carcinoma | 0.750 | Provisional weight based on Dutch weights |
| Terminal stage | 0.930 | Dutch weight for end-stage disease |
| 16. Prostate cancer |  |  |
| Diagnostic, primary therapy, localized cancer | 0.270 | Dutch weight |
| Follow-up without active therapy (watchful waiting) | 0.270 | Dutch weight |
| In remission | 0.200 | Dutch weight |
| Clinically disease-free after primary therapy | 0.180 | Dutch weight |
| Hormone refractory cancer | 0.640 | Dutch weight |
| Terminal stage | 0.930 | Dutch weight end-stage disease |
| 17. Testicular cancer |  |  |
| Diagnosis and primary therapy | 0.270 | Provisional weight based on Dutch weights |
| State after intentionally curative primary therapy | 0.18 | Provisional weight based on Dutch weights |
| In remission | 0.180 | Provisional weight based on Dutch weights |
| Disseminated carcinoma | 0.640 | Provisional weight based on Dutch weights |
| Terminal stage | 0.930 | Dutch weight for end-stage disease |
| 18. Bladder cancer |  |  |
| Diagnosis and primary therapy | 0.270 | Provisional weight based on Dutch weights |
| State after intentionally curative primary therapy | 0.18 | Provisional weight based on Dutch weights |
| In remission | 0.180 | Provisional weight based on Dutch weights |
| Disseminated carcinoma | 0.640 | Provisional weight based on Dutch weights |
| Terminal stage | 0.930 | Dutch weight for end-stage disease |
| 19. Kidney cancer |  |  |
| Diagnosis and primary therapy | 0.270 | Provisional weight based on Dutch weights |
| State after intentionally curative primary therapy | 0.18 | Provisional weight based on Dutch weights |
| In remission | 0.180 | Provisional weight based on Dutch weights |
| Disseminated carcinoma | 0.640 | Provisional weight based on Dutch weights |
| Terminal stage | 0.930 | Dutch weight for end-stage disease |
| 20. Brain cancer |  |  |
| Diagnosis and primary therapy | 0.680 | Provisional weight based on Dutch weights |
| State after intentionally curative primary therapy | 0.18 | Provisional weight based on Dutch weights |
| Disseminated carcinoma | 0.750 | Provisional weight based on Dutch weights |
| Terminal stage | 0.930 | Dutch weight for end-stage disease |
| 21. Thyroid cancer |  |  |
| Diagnosis and primary therapy | 0.270 | Provisional weight based on Dutch weights |
| State after intentionally curative primary therapy | 0.18 | Provisional weight based on Dutch weights |
| In remission | 0.180 | Provisional weight based on Dutch weights |
| Disseminated carcinoma | 0.640 | Provisional weight based on Dutch weights |
| Terminal stage | 0.930 | Dutch weight for end-stage disease |
| Low grade, dissemination stage I and II | 0.190 | Dutch weight |
| Low grade, dissemination stage III and IV | 0.610 | Dutch weight |
| Intermediate/high grade, dissemination stage I | 0.550 | Dutch weight |
| Intermediate/high grade, dissemination stage II, III or IV | 0.750 | Dutch weight |
| Temporary remission after treatment | 0.190 | Dutch weight |
| Preterminal phase | 0.750 | Dutch weight |
| Terminal phase | 0.930 | Dutch weight for end-stage disease |
| Complete remission | 0.190 | Dutch weight |
| 22b. Hodgkin’s disease |  |  |
| Low grade, dissemination stage I and II | 0.190 | Dutch weight |
| Low grade, dissemination stage III and IV | 0.610 | Dutch weight |
| Intermediate/high grade, dissemination stage I | 0.550 | Dutch weight |
| Intermediate/high grade, dissemination stage II, III or IV | 0.750 | Dutch weight |
| Temporary remission after treatment | 0.190 | Dutch weight |
| Preterminal phase | 0.750 | Dutch weight |
| Terminal phase | 0.930 | Dutch weight for end-stage disease |
| Complete remission | 0.190 | Dutch weight |
| 23. Multiple myeloma |  |  |
| Diagnosis and primary therapy | 0.190 | Provisional weight based on Dutch weights |
| State after intentionally curative primary therapy | 0.190 | Provisional weight based on Dutch weights |
| In remission | 0.190 | Provisional weight based on Dutch weights |
| Disseminated carcinoma | 0.750 | Provisional weight based on Dutch weights |
| Terminal stage | 0.930 | Dutch weight for end-stage disease |
| 24a. Acute myeloid leukemia |  |  |
| Diagnosis and primary therapy | 0.550 | Provisional weight based on Dutch weights |
| State after intentionally curative primary therapy | 0.19 | Provisional weight based on Dutch weights |
| Preterminal stage | 0.750 | Provisional weight based on Dutch weights |
| Terminal stage | 0.930 | Dutch weight for end-stage disease |
| 24b. Chronic myeloid leukemia |  |  |
| Diagnosis and primary therapy | 0.550 | Provisional weight based on Dutch weights |
| State after intentionally curative primary therapy | 0.19 | Provisional weight based on Dutch weights |
| In remission | 0.190 | Provisional weight based on Dutch weights |
| Preterminal stage | 0.750 | Provisional weight based on Dutch weights |
| Terminal stage | 0.930 | Dutch weight for end-stage disease |
| 24c. Acute lymphoid leukemia | 0.19 |  |
| Diagnosis and primary therapy | 0.550 | Provisional weight based on Dutch weights |
| State after intentionally curative primary therapy | 0.19 | Provisional weight based on Dutch weights |
| In remission | 0.190 | Provisional weight based on Dutch weights |
| Preterminal stage | 0.750 | Provisional weight based on Dutch weights |
| Terminal stage | 0.930 | Dutch weight for end-stage disease |
| 24d. Chronic lymphoid leukemia | 0.19 |  |
| Diagnosis and primary therapy | 0.550 | Provisional weight based on Dutch weights |
| State after intentionally curative primary therapy | 0.19 | Provisional weight based on Dutch weights |
| In remission | 0.190 | Provisional weight based on Dutch weights |
| Preterminal stage | 0.750 | Provisional weight based on Dutch weights |
| Terminal stage | 0.930 | Dutch weight for end-stage disease |
| 1. Uterine myomas |  |  |
| Symptomatic cases | 0.066 | Estimated using EQ-5D+ regression model |
| Hysterectomy or myomectomy | 0.349 | Estimated using EQ-5D+ regression model |
| Reproductive disability | 0.180 | GBD weight for infertility |
| 2. Benign brain tumor |  |  |
| Diagnosis and primary therapy | 0.680 | Provisional weight based on Dutch weights |
| State after intentionally curative primary | 0.180 | Provisional weight based on Dutch weights |
| Pre-terminal stage | 0.750 | Provisional weight based on Dutch weights |
| Terminal stage | 0.930 | Dutch weight for end-stage disease |
| **H. Diabetes mellitus** |  |  |
| 1. Type 1 diabetes |  |  |
| Cases | 0.070 | Dutch weight |
| Retinopathy—moderate vision loss | 0.170 | Dutch weight |
| Retinopathy—severe vision loss | 0.430 | Dutch weight |
| Cataract—mild vision loss | 0.020 | Dutch weight |
| Cataract—moderate vision loss | 0.170 | Dutch weight |
| Cataract—severe vision loss | 0.430 | Dutch weight |
| Glaucoma—mild vision loss | 0.020 | Dutch weight |
| Glaucoma—moderate vision loss | 0.170 | Dutch weight |
| Glaucoma—severe vision loss | 0.430 | Dutch weight |
| Neuropathy | 0.190 | Dutch weight |
| Nephropathy | 0.290 | Dutch weight |
| Diabetic foot | 0.220 | GBD weight |
| Amputation—toe | 0.064 | GBD weight |
| Amputation—foot or leg | 0.300 | GBD weight |
| 2. Type 2 diabetes |  |  |
| Cases | 0.070 | Dutch weight |
| Retinopathy—moderate vision loss | 0.170 | Dutch weight |
| Retinopathy—severe vision loss | 0.430 | Dutch weight |
| Cataract—mild vision loss | 0.020 | Dutch weight |
| Cataract—moderate vision loss | 0.170 | Dutch weight |
| Cataract—severe vision loss | 0.430 | Dutch weight |
| Glaucoma—mild vision loss | 0.020 | Dutch weight |
| Glaucoma—moderate vision loss | 0.170 | Dutch weight |
| Glaucoma—severe vision loss | 0.430 | Dutch weight |
| Neuropathy | 0.190 | Dutch weight |
| Nephropathy | 0.290 | Dutch weight |
| Diabetic foot | 0.220 | GBD weight |
| Amputation—toe | 0.064 | GBD weight |
| Amputation—foot or leg | 0.300 | GBD weight |
| **I. Endocrine and metabolic disorders** |  |  |
| 1. Non-deficiency anemia |  |  |
| a. Thalassemia |  |  |
| Very severe anemia | 0.250 | GBD weight |
| b. Other non-deficiency anemia |  |  |
| Genetically inherited anemias | 0.090 | GBD weight |
| Severe anemia | 0.090 | GBD weight |
| Very severe anemia | 0.250 | GBD weight |
| 2. Cystic fibrosis |  |  |
| Cases | 0.530 |  |
| 3. Hemophilia |  |  |
| Severe cases | 0.270 | Weight based on QALY measurements |
| Moderate cases | 0.050 | Weight based on QALY measurements |
| **J. Mental disorders** |  |  |
| 1. Substance use disorders |  |  |
| a. Alcohol dependence and harmful use |  |  |
| Harmful use | 0.110 | Dutch weight for problem drinking |
| Moderate dependence | 0.330 | Average of Dutch weights for problem drinking and manifest alcoholism |
| Manifest alcoholism | 0.550 | Dutch weight |
| b. Heroin or polydrug dependence and harmful use |  |  |
| Cases | 0.270 | Locally derived weight, slightly higher than GBD weight 0.252 |
| c. Benzodiazepine dependence and harmful use |  |  |
| Cases | 0.184 | Extrapolation by Australian mental health experts |
| d. Cannabis dependence and harmful use |  |  |
| Cases | 0.113 | Extrapolation by Australian mental health experts |
| e. Other drug dependence and harmful use |  |  |
| Stimulant dependence and harmful use | 0.110 | Dutch weight for problem drinking |
| Other drug dependence | 0.113 | Dutch weight for cannabis dependence |
| Analgesic nephropathy | 0.290 | Dutch weight for diabetic nephropathy |
| 2. Schizophrenia |  |  |
| Cases | 0.434 | Composite GBD weight—psychosis (30%), treated |
| 3. Affective disorders |  |  |
| a. Major depression |  |  |
| Dysthymia cases | 0.140 | Dutch weight for mild depression |
| Major depressive episode—mild | 0.140 | Dutch weight |
| Major depressive episode—moderate | 0.350 | Dutch weight |
| Major depressive episode—severe | 0.760 | Dutch weight |
| b. Bipolar affective disorder |  |  |
| Cases | 0.176 | Composite Dutch weight - mild depression (50%) non episodes; 25% moderate depression, 25% local extrapolated weight for episodic manic phase |
| 4. Anxiety disorders |  |  |
| a. Panic disorder |  |  |
| Mild to moderate panic disorder | 0.160 | Dutch weight |
| Severe panic disorder | 0.690 | Dutch weight |
| b. Agoraphobia |  |  |
| Mild to moderate agoraphobia | 0.110 | Dutch weight |
| Severe agoraphobia | 0.550 | Dutch weight |
| c. Social phobia |  |  |
| Mild to moderate social phobia | 0.170 | Dutch weight |
| Severe social phobia | 0.590 | Dutch weight |
| d. Generalized anxiety disorder (GAD) |  |  |
| Mild to moderate GAD | 0.170 | Dutch weight |
| Severe GAD | 0.600 | Dutch weight |
| e. Obsessive-compulsive disorder (OCD) |  |  |
| Mild to moderate OCD | 0.170 | Dutch weight |
| Severe OCD | 0.600 | Dutch weight |
| f. Post-traumatic stress disorder (PTSD) |  |  |
| Mild to moderate PTSD | 0.130 | Dutch weight |
| Severe PTSD | 0.510 | Dutch weight |
| g. separation anxiety disorder |  |  |
| Mild to moderate separation anxiety disorder | 0.110 | Dutch weight for mild to moderate agoraphobia |
| Severe separation anxiety disorder | 0.550 | Dutch weight for severe agoraphobia |
| 5. Borderline personality disorder |  |  |
| Symptomatic cases | 0.540 | Extrapolation by Australian mental health experts |
| 6. Eating disorders |  |  |
| a. Anorexia nervosa |  |  |
| Cases | 0.280 | Dutch weight |
| b. Bulimia nervosa |  |  |
| Cases | 0.280 | Dutch weight |
| 7. Childhood conditions |  |  |
| a. Attention-deficit hyperactivity disorder |  |  |
| Mild | 0.020 | Dutch weight. |
| Moderate to severe | 0.150 | Dutch weight. |
| b. Autism and Asperger’s syndrome |  |  |
| Autism cases | 0.550 | Dutch weight |
| Asperger’s syndrome cases | 0.250 | Average of Dutch weights for moderate/severe ADHD and for autism |
| 8. Mental retardation (no defined etiology) |  |  |
| Mild intellectual disability | 0.290 | Dutch weight |
| Moderate intellectual disability | 0.430 | Dutch weight |
| Severe intellectual disability | 0.820 | Dutch weight |
| Profound intellectual disability | 0.760 | Dutch weight |
| **K. Nervous system and sense organ disorder** |  |  |
| 1. Dementia |  |  |
| Mild | 0.270 | Dutch weight |
| Moderate | 0.630 | Dutch weight |
| Severe | 0.940 | Dutch weight |
| 2. Epilepsy |  |  |
| Epilepsy | 0.110 | Dutch weight |
| 3. Parkinson’s’ disease |  |  |
| Initial stage | 0.480 | Dutch weight |
| Intermediate stage | 0.790 | Dutch weight |
| End-stage | 0.920 | Dutch weight |
| 4. Multiple sclerosis |  |  |
| Relapsing-remitting phase | 0.330 | Dutch weight |
| Progressive phase | 0.670 | Dutch weight |
| Progressive from onset | 0.670 | Dutch weight |
| 5. Motor neuron disease |  |  |
| Cases | 0.670 | Dutch weight for progressive phase of multiple sclerosis. |
| 6. Huntington’s chorea |  |  |
| Initial stage | 0.480 | Dutch weight for initial stage Parkinson’s disease |
| Intermediate stage | 0.790 | Dutch weight for intermediate stage Parkinson’s disease |
| End-stage | 0.920 | Dutch weight for end-stage Parkinson’s disease |
| 7. Muscular dystrophy |  |  |
| Initial stage | 0.480 | Dutch weight for initial stage Parkinson’s disease |
| Paraplegia | 0.570 | Dutch weight |
| Quadriplegia | 0.840 | Dutch weight |
| 8. Sense organ disorders |  |  |
| a. Glaucoma |  |  |
| Cases | 0.000 | GBD and Dutch weights |
| Mild vision loss | 0.020 | Dutch weight |
| Moderate vision loss | 0.170 | Dutch weight |
| Severe vision loss | 0.430 | Dutch weight |
| b. Cataracts |  |  |
| Cases | 0.000 | GBD and Dutch weights |
| Mild vision loss | 0.020 | Dutch weight |
| Moderate vision loss | 0.170 | Dutch weight |
| Severe vision loss | 0.430 | Dutch weight |
| c. Age-related vision disorders |  |  |
| Mild vision loss | 0.020 | Dutch weight |
| Moderate vision loss | 0.170 | Dutch weight |
| Severe vision loss | 0.430 | Dutch weight |
| d. Adult-onset hearing loss |  |  |
| Mild hearing loss (25–34 dBHTL) | 0.020 | One half of Dutch weight for mild hearing loss |
| Mild hearing loss (35–44 dBHTL) | 0.040 | Dutch weight |
| Moderate hearing loss | 0.120 | Dutch weight |
| Severe hearing loss | 0.370 | Dutch weight |
| **L. Cardiovascular disease** |  |  |
| 1. Rheumatic heart disease |  |  |
| Rheumatic fever | 0.047 | Regression weight for influenza |
| Rheumatic heart disease |  |  |
| Untreated | 0.323 | GBD weight |
| Treated | 0.171 | GBD weight |
| 2. Ischemic heart disease |  |  |
| Angina pectoris | 0.178 | Dutch weight |
| Acute myocardial infarction | 0.395 | GBD (treated) age-specific weights (average shown here) |
| Heart failure | 0.353 | Dutch weight |
| 3. Stroke |  |  |
| First- ever stroke with full recovery | 0.000 |  |
| Mild permanent impairments | 0.360 | Dutch weight |
| Moderate permanent impairments | 0.630 | Dutch weight |
| Severe permanent impairments | 0.920 | Dutch weight |
| 4. Inflammatory heart disease |  |  |
| Cardiomyopathy cases | 0.353 | Dutch weight for heart failure |
| Endocarditis cases | 0.353 | Dutch weight for heart failure |
| Myocarditis cases | 0.353 | Dutch weight for heart failure |
| Pericarditis cases | 0.353 | Dutch weight for heart failure |
| 5. Hypertensive heart disease |  |  |
| Cases | 0.352 | Based on Dutch weight for heart failure |
| 6. Non-rheumatic valvular disease |  |  |
| cases | 0.060 | Dutch weight for mild heart failure |
| 7. Aortic aneurysm |  |  |
| Cases | 0.430 | Dutch weight for early colorectal cancer |
| 8. Peripheral arterial disease |  |  |
| Cases | 0.248 | Estimated using EQ-5D+ regression model |
| Amputation | 0.209 | GBD weight |
| **M. Chronic respiratory disease** |  |  |
| 1. Chronic obstructive pulmonary disease (COPD) |  |  |
| Mild to moderate COPD | 0.170 | Dutch weight |
| Severe COPD | 0.530 | Dutch weight |
| 2. Asthma |  |  |
| Mild asthma | 0.030 | Dutch weight |
| Severe asthma | 0.230 | Estimated using EQ-5D+ regression model and |
|  |  | Australian data on severity distribution of disability |
| 3. Other chronic respiratory diseases | 0.164 | Provisional weight—average weight for COPD |
| **N. Diseases of the digestive system** |  |  |
| 1. Peptic ulcer disease | 0.066 | Dutch weight |
| 2. Cirrhosis of the liver | 0.339 | GBD weight |
| 3. Appendicitis | 0.463 | GBD weight |
| 4. Intestinal obstruction |  |  |
| Cases | 0.463 | Dutch weight for appendicitis |
| Stoma closed | 0.211 | Estimated using EQ-5D+ regression model |
| Stoma continuing | 0.211 | Estimated using EQ-5D+ regression model |
| 5. Diverticulitis |  |  |
| Cases | 0.400 | Dutch weight for inflammatory bowel disease—active exacerbation |
| Stoma closed | 0.211 | Estimated using EQ-5D+ regression model |
| Stoma continuing | 0.211 | Estimated using EQ-5D+ regression model |
| 6. Gall bladder and bile duct disease |  |  |
| Cases | 0.349 | Estimated using EQ-5D+ regression model |
| 7. Pancreatitis |  |  |
| Cases | 0.349 | Estimated using EQ-5D+ regression model |
| 8. Inflammatory bowel disease |  |  |
| Crohn’s disease | 0.224 | Dutch weight |
| Ulcerative colitis | 0.224 | Dutch weight |
| Stoma closed | 0.211 | Estimated using EQ-5D+ regression model |
| Stoma continuing | 0.211 | Estimated using EQ-5D+ regression model |
| 9. Vascular insufficiency of intestine |  |  |
| Cases | 0.400 | Dutch weight for inflammatory bowel disease—active |
|  |  | exacerbation |
| Stoma closed | 0.211 | Estimated using EQ-5D+ regression model |
| Stoma continuing | 0.211 | Estimated using EQ-5D+ regression model |
| **O. Genitourinary diseases** |  |  |
| 1. Nephritis and nephrosis |  |  |
| End-stage renal failure with dialysis | 0.290 | Dutch weight for diabetic nephropathy |
| End-stage renal failure with transplant | 0.290 | Dutch weight for diabetic nephropathy |
| Transplanted patient | 0.110 | GBD weight for treated renal failure, Dutch weight for uncertain prognosis |
| Untreated end-stage renal failure | 0.104 | GBD weight |
| 2. Benign prostatic hypertrophy |  |  |
| Symptomatic case | 0.038 | GBD weight |
| Prostatectomy | 0.349 | Estimated using EQ-5D+ regression model |
| Urethral stricture | 0.151 | GBD weight |
| Impotence | 0.195 | GBD weight |
| Severe urinary incontinence | 0.157 | Estimated using EQ-5D+ regression model |
| 3. Urinary incontinence |  |  |
| Occasional urine leakage | 0.000 | No weight for occasional urine leakage |
| Moderate incontinence | 0.025 | GBD weight for stress incontinence (0.033 for 60+) |
| Severe incontinence | 0.157 | Estimated using EQ-5D+ regression model |
| 4. Other genitourinary diseases |  |  |
| Menstrual disorders | 0.033 | Estimated from EQ-5D+ regression model |
| Hysterectomy | 0.349 | Estimated from EQ-5D+ regression model |
| Reproductive disability following hysterectomy |  |  |
| for hemorrhagia | 0.180 | Estimated from EQ-5D+ regression model |
| for genital prolapse | 0.180 | Estimated from EQ-5D+ regression model |
| for endometriosis | 0.180 | Estimated from EQ-5D+ regression model |
| Other short-term reproductive disability | 0.180 | GBD weight |
| Other long-term reproductive disability | 0.180 | GBD weight |
| **P. Skin diseases** |  |  |
| 1. Eczema | 0.056 | Estimated from EQ-5D+ regression model |
| 2. Other skin diseases | 0.056 | Estimated from EQ-5D+ regression model |
| **Q. Musculoskeletal diseases** |  |  |
| 1. Rheumatoid arthritis |  |  |
| Mild | 0.210 | Dutch weight |
| Moderate | 0.370 | Dutch weight |
| Severe | 0.940 | Dutch weight |
| 2. Osteoarthritis |  |  |
| Grade 2 (radiological) hip or knee (asympt.) | 0.010 | Dutch weight |
| Grade 2 symptomatic | 0.140 | Dutch weight |
| Grade 3–4 (radiological) hip or knee (asympt.) | 0.140 | Dutch weight |
| Grade 3–4 symptomatic | 0.420 | Dutch weight |
| 3. Chronic back pain |  |  |
| Episodes | 0.060 | Dutch weight |
| 4. Slipped disc |  |  |
| Episodes | 0.060 | Dutch weight for back problems |
| Excision or destruction of disc | 0.060 | Dutch weight for back problems |
| Chronic pain | 0.125 | Estimated using EQ-5D+ regression model |
| 5. Occupational overuse syndrome |  |  |
| Mild handicap or disability | 0.056 | Estimated using EQ-5D+ regression model |
| Moderate handicap | 0.293 | Estimated using EQ-5D+ regression model |
| Severe or profound handicap | 0.516 | Estimated using EQ-5D+ regression model |
| 6. Osteoporosis |  |  |
| Diagnosed cases | 0.009 | Estimated using EQ-5D+ regression model |
| 7. Other musculoskeletal disorders |  |  |
| Recent non-chronic episodes | 0.060 | Dutch weight for low back pain |
| Chronic conditions | 0.060 | Dutch weight for low back pain |
| **R. Congenital anomalies** |  |  |
| 1. Anencephaly |  |  |
| Live born cases | 1.000 |  |
| 2. Spina bifida |  |  |
| Low-level spina bifida aperta | 0.160 | Dutch weight |
| Medium-level spina bifida aperta | 0.500 | Dutch weight |
| High-level spina bifida aperta | 0.680 | Dutch weight |
| 3. Congenital heart disease |  |  |
| Surgically treated congenital atrial or | 0.030 | Dutch weight |
| ventricular septal defect |  |  |
| Child/adolescent in permanent stage after | 0.200 | Dutch weight |
| surgical treatment for Fallot's tetralogy |  |  |
| or transposition of great arteries |  |  |
| Young adult in permanent stage after surgical | 0.110 | Dutch weight |
| treatment for Fallot's tetralogy or |  |  |
| transposition of great arteries |  |  |
| Child/adolescent in permanent stage after | 0.020 | Dutch weight |
| surgical treatment for pulmonary |  |  |
| stenosis |  |  |
| Young adult in permanent stage after surgical | 0.160 | Dutch weight |
| treatment for pulmonary stenosis |  |  |
| Complex not curatively operable congenital | 0.720 | Dutch weight |
| heart disease |  |  |
| 4. Cleft lip and/or palate |  |  |
| Cleft palate—untreated | 0.231 | GBD weight |
| Cleft palate—treated | 0.015 | GBD weight |
| Cleft lip—untreated | 0.098 | GBD weight |
| Cleft lip—treated | 0.016 | GBD weight |
| 5. Digestive system malformations |  |  |
| a. Anorectal atresia |  |  |
| Cases | 0.850 | GBD weight for anorectal atresia |
| Long-term disability | 0.037 | GBD weight for symptomatic urethritis |
| b. Esophageal atresia |  |  |
| Cases | 0.850 | GBD weight for anorectal atresia |
| Long-term disability | 0.037 | GBD weight for symptomatic urethritis |
| c. Other digestive system malformations |  |  |
| Small intestine atresia | 0.850 | GBD weight for digestive system atresias |
| Other | 0.850 | GBD weight for digestive system atresias |
| 6. Urogenital tract malformations |  |  |
| a. Renal agenesis |  |  |
| Bilateral renal agenesis or dysgenesis | 0.850 | GBD weight for renal agenesis |
| Unilateral renal agenesis or dysgenesis | 0.037 | GBD weight for symptomatic urethritis |
| End-stage renal failure | 0.294 | Dutch weight |
| b. Other urogenital tract malformations |  |  |
| Hypospadias | 0.000 | Assumed negligible ongoing disability |
| Cystic kidney disease | 0.037 | GBD weight for acute urethritis |
| Obstructive defects of renal pelvis and ureter | 0.037 | GBD weight for renal diseases |
| Other urinary tract malformations | 0.290 | Dutch weight for renal failure |
| 7. Abdominal wall defect |  |  |
| Cases | 0.850 | GBD weight for abdominal wall defect |
| Long-term disability | 0.200 | Dutch weight for permanent stage treated CVD malformation |
| 8. Down syndrome |  |  |
| Child aged 0–9 with other malformations | 0.690 | Dutch weight |
| Child aged 0–9 without other malformations | 0.510 | Dutch weight |
| Person aged 10–39 years | 0.350 | Dutch weight |
| Adult 40 years of age and over | 0.650 | Dutch weight |
| 9. Other chromosomal conditions |  |  |
| Mild intellectual disability | 0.290 | Dutch weight |
| Moderate intellectual disability | 0.430 | Dutch weight |
| Severe intellectual disability | 0.820 | Dutch weight |
| Profound intellectual disability | 0.760 | Dutch weight |
| **S. Oral health** |  |  |
| 1. Dental caries |  |  |
| Episode resulting in filling | 0.005 | Dutch weight |
| Episode resulting in tooth loss | 0.014 | Estimated using EQ-5D+ regression model |
| 2. Periodontal disease |  |  |
| Gingivitis | 0.000 | Dutch weight |
| Pockets 6 mm or more deep | 0.001 | Dutch weight |
| 3. Edentulism |  |  |
| Cases | 0.004 | Estimated using EQ-5D+ regression model |
| **V. Ill-defined conditions** |  |  |
| 1. Chronic fatigue syndrome |  |  |
| Mild handicap | 0.137 | Estimated using EQ-5D+ regression model |
| Moderate handicap | 0.449 | Estimated using EQ-5D+ regression model |
| Severe or profound handicap | 0.760 | Estimated using EQ-5D+ regression model |
| **III. Injuries - type of injury sequelae** |  |  |
| 1. Fractures |  |  |
| Skull—short-term | 0.431 | GBD weight |
| Skull—long-term | 0.350 | GBD weights (0.404 for ages 65+) |
| Face bones | 0.223 | GBD weight |
| Vertebral column | 0.266 | GBD weight |
| Rib or sternum | 0.199 | GBD weight |
| Pelvis | 0.247 | GBD weight |
| Clavicle, scapula or humerus | 0.153 | GBD weight |
| Radius or ulna | 0.180 | GBD weight |
| Hand bones | 0.100 | GBD weight |
| Femur—short-term | 0.372 | GBD weight |
| Femur—long-term | 0.272 | GBD weight |
| Patella, tibia or fibula | 0.271 | GBD weight |
| Ankle | 0.196 | GBD weight |
| Foot bones | 0.077 | GBD weight |
| 2. Injured spinal cord | 0.725 | GBD weight |
| 3. Dislocations |  |  |
| Shoulder, elbow or hip | 0.074 | GBD weight |
| Other dislocation | 0.074 | GBD weight for shoulder, elbow or hip dislocation |
| 4. Sprains | 0.064 | GBD weight |
| 5. Intracranial injuries |  |  |
| Short-term | 0.359 | GBD weight |
| Long-term | 0.350 | GBD weight |
| 6. Internal injuries | 0.208 | GBD weight |
| 7. Open wound | 0.108 | GBD weight |
| 8. Injury to eyes |  |  |
| Short-term | 0.108 | GBD weight for open wound |
| Long-term | 0.298 | GBD weight (0.301 for ages 0–14) |
| 9. Amputations |  |  |
| Thumb | 0.165 | GBD weight |
| Finger | 0.102 | GBD weight |
| Arm | 0.257 | GBD weight |
| Toe | 0.102 | GBD weight |
| Foot | 0.300 | GBD weight |
| Leg | 0.300 | GBD weight |
| 10. Crushing | 0.218 | GBD weight |
| 11. Burns |  |  |
| Less than 20%—short-term | 0.158 | GBD weight |
| Less than 20%—long-term | 0.001 | GBD weight |
| 20 to 60%—short-term | 0.441 | GBD weight |
| 20 to 60%—long-term | 0.255 | GBD weight |
| Greater than 60%—short-term | 0.441 | GBD weight |
| Greater than 60%—long-term | 0.255 | GBD weight |
| 12. Injured nerves |  |  |
| Short-term | 0.064 | GBD weight |
| Long-term | 0.064 | GBD weight |
| 13. Poisoning | 0.608 | GBD weight (0.611 for ages 0–14) |
| **T. Unintentional injuries** |  |  |
| 1. Road traffic accidents | 0.149 | Average weight across all injury sequelae |
| 2. Other transport accidents | 0.142 | Average weight across all injury sequelae |
| 3. Poisoning | 0.593 | Average weight across all injury sequelae |
| 4. Falls | 0.141 | Average weight across all injury sequelae |
| 5. Fires/burns/scalds | 0.172 | Average weight across all injury sequelae |
| 6. Drowning | 0.211 | Average weight across all injury sequelae |
| 7. Sports injuries | 0.118 | Average weight across all injury sequelae |
| 8. Natural and environmental factors | 0.158 | Average weight across all injury sequelae |
| 9. Machinery accidents | 0.112 | Average weight across all injury sequelae |
| 10. Suffocation and foreign bodies | 0.162 | Average weight across all injury sequelae |
| 11. Adverse effects of medical treatment | 0.433 | Average weight across all injury sequelae |
| a. Surgical and medical misadventure | 0.38 | Average weight across all injury sequelae |
| b. Adverse effects of drugs in therapeutic use | 0.453 | Average weight across all injury sequelae |
| 12. Other unintentional injuries | 0.112 | Average weight across all injury sequelae |
| a. Cutting and piercing accidents | 0.10 | Average weight across all injury sequelae |
| b. Striking and crushing accidents | 0.157 | Average weight across all injury sequelae |
| c. Other unintentional injuries | 0.11 | Average weight across all injury sequelae |
| **U. Intentional injuries** |  |  |
| 1. Suicide and self-inflicted injuries | 0.447 | Average weight across all injury sequelae |
| 2. Homicide and violence | 0.166 | Average weight across all injury sequelae |
| 3. Legal intervention and war | 0.12 | Average weight across all injury sequelae |
| **Disease category, subcategory, or sequelae with disability weights assigned by Iranian burden of disease study team** | **Disability weight** | **ICD Code** |
| Typhoid fever | 0.2 | A01 |
| Amoebiasis | 0.086 | A06 |
| Giardiasis | 0.008 | A07.1 |
| Cutaneous anthrax | 0.046 | A22 |
| Brucellosis | 0.23 | A23 |
| Trichomoniasis | 0.067 | A59 |
| Anogenital herpesviral infection (genital herpes) | 0.067 | A60 |
| Anogenital (venereal) warts | 0.067 | A63.0 |
| Crimean-Congo Hemorrhagic Fever | 0.613 | A98.0 |
| Varicella without complication | 0.13 | B01.9 |
| Mumps | 0.13 | B26 |
| Hydatid cyst | 0.1 | B67 |
| Teniasis | 0.002 | B68 |
| Hymenolepiasis | 0.001 | B71.0 |
| Ascariasis | 0.001 | B77 |
| Trichuriasis | 0.001 | B79 |
| Enterobiasis | 0.01 | B80 |
| Trichostrongyliasis | 0.001 | B81.2 |
| Benign neoplasm of breast (fibroadenoma and fibrocystic disease) | 0.1 | D24 |
| Leiomyoma of uterus | 0.09 | D25 |
| G6PD deficiency | 0.011 | D55.0 |
| Idiopathic thrombocytopenic purpura | 0.152 | D69.3 |
| Congenital hypothyroidism with diffuse goiter | 0.65 | E03.0 |
| Congenital hypothyroidism without diffuse goiter | 0.35 | E03.1 |
| Polycystic ovarian syndrome | 0.04 | E28.2 |
| Rickets | 0.108 | E55.0 |
| Phenylketonuria, classical | 0.65 | E70.0 |
| Other hyperphenylalaninaemias | 0.35 | E70.1 |
| Carpal tunnel syndrome | 0.1 | G 56.0 |
| **Disease category, subcategory, or sequelae with disability weights assigned by Iranian burden of disease study team (continued)** |  | **ICD Code** |
| Tension-type headache | 0.05 | G44.2 |
| Conjunctivitis | 0.152 | H10 |
| Disorders of refraction | 0.03 | H52 |
| Mitral valve prolapse | 0.06 | I34.1 |
| Gastro-esophageal reflux disease | 0.1 | K21 |
| Dyspepsia | 0.1 | K30 |
| Inguinal hernia | 0.463 | K40 |
| Constipation | 0.01 | K59.0 |
| Celiac disease | 0.06 | K90.0 |
| Pilonidal cyst | 0.03 | L05 |
| Pemphigus | 0.2 | L10 |
| Atopic dermatitis | 0.056 | L20 |
| Seborrhoeic dermatitis | 0.01 | L21 |
| Contact dermatitis | 0.01 | L23-L25 |
| Psoriasis | 0.08 | L40 |
| Lichen planus | 0.05 | L43 |
| Alopecia areata | 0.01 | L63 |
| Acne vulgaris | 0.018 | L70.0 |
| Vitiligo | 0.02 | L80 |
| Arthrosis of neck | 0.04 | M19 |
| Systemic lupus erythematosus | 0.2 | M32 |
| Lumbago with sciatica | 0.135 | M54.4 |
| Lumbago without sciatica (low back pain) | 0.04 | M54.5 |
| Frozen shoulder | 0.16 | M75.0 |
| Rotator cuff syndrome | 0.065 | M75.1 |
| Medial epicondylitis | 0.0325 | M77.0 |
| Lateral epicondylitis | 0.0375 | M77.1 |
| Calculus of kidney and ureter | 0.056 | N20.0, N20.1 |
| Acute cystitis | 0.1 | N30.0 |
| **Disease category, subcategory, or sequelae with disability weights assigned by Iranian burden of disease study team (continued)** |  | **ICD Code** |
| Nongonococcal urethritis | 0.067 | N34.1 |
| Infertility due to mumps | 0.3 | N51.1 |
| Endometriosis | 0.1 | N80 |
| Female genital prolapse | 0.1 | N81 |
| Menorrhagia | 0.09 | N92.0 |
| Premenstrual tension syndrome | 0.1 | N94.3 |
| Menopausal and female climacteric states | 0.1 | N95.1 |
| Postmenopausal atrophic vaginitis | 0.03 | N95.2 |
| Pregnancy | 0.1 | - |
| Ectopic pregnancy | 0.463 | O00 |
| Hydatidiform mole | 0.18 | O01 |
| Spontaneous abortion | 0.18 | O03 |
| Threatened abortion | 0.1 | O20.0 |
| Excessive vomiting in pregnancy | 0.2 | O21 |
| Pyelonephritis in pregnancy | 0.18 | O23.0 |
| Placenta previa (without severe hemorrhage) | 0.1 | O44.1 |
| Placenta previa (without severe hemorrhage) | 0.5 | O44.1 |
| Single delivery (para 1, 2, or 3) | 0.2 | O80, O81, O83 |
| Stillbirth | 0.3 | O80-O84 |
| Single delivery by caesarean section | 0.3 | O82 |
| Puerperal sepsis | 0.613 | O85 |
| Abscess of breast / mastitis associated with childbirth | 0.2 | O91.1 |
| Hirschsprung's disease | 0.25 | Q43.1 |
| Hypospadias | 0.033 | Q54 |
| Polycystic kidney, adult type | 0.063 | Q61.2 |
| Congenital vesico-uretero-renal reflux | 0.051 | Q62.7 |

Appendix table S2. Top 11 disease and injury causes with the highest DALY rates per 100,000 by age groups; both sexes, Iran 2003

| **0-4 years** | | **5-14 years** | | **15-44 years** | | **45-59 years** | | **60+ years** | |
| --- | --- | --- | --- | --- | --- | --- | --- | --- | --- |
| **Cause** | **DALY**  **Rate** | **Cause** | **DALY**  **Rate** | **Cause** | **DALY**  **Rate** | **Cause** | **DALY**  **Rate** | **Cause** | **DALY**  **Rate** |
| Prematurity with appropriate birth weight | 2237 | Transport accidents | 798 | Transport accidents | 5367 | Ischemic heart disease ^(1)^ | 3820 | Ischemic heart disease ^(1)^ | 23622 |
| Perinatal respiratory and cardiovascular^(2)^ | 1939 | Anemias ^(3)^ | 345 | Addiction ^(4)^ | 2922 | Knee osteoarthrosis | 1746 | Falls | 16374 |
| LBW + prematurity ^(5)^ | 1848 | Diarrheal disease | 274 | Natural disasters ^(6)^ | 2797 | Depression ^(7)^ | 1601 | Cerebrovascular diseases | 11387 |
| Complications of pregnancy, labor and delivery ^(8)^ | 1090 | URI ^(9)^ and otitis media | 249 | Depression ^(7)^ | 2744 | Transport accidents | 1557 | COPD ^(10)^ | 5969 |
| Transport accidents | 739 | Asthma | 205 | Ischemic heart disease ^(1)^ | 2322 | Cerebrovascular diseases | 1438 | Transport accidents | 4799 |
| Congenital heart disease | 707 | Epilepsy | 188 | Low back pain | 1434 | Menopause ^(11)^ | 1019 | Dementia | 4161 |
| Birth trauma | 704 | Burns | 179 | Bipolar disorders ^(12)^ | 1150 | Low back pain | 952 | Stomach cancer | 2740 |
| Congenital hypothyroidism | 664 | Falls | 160 | Knee osteoarthritis | 1087 | Diabetes mellitus | 840 | Depression ^(7)^ | 2672 |
| Infections specified to the perinatal period | 580 | Depression ^(7)^ | 140 | Exposure to mechanical forces | 1065 | Falls | 799 | Cataract | 2643 |
| Down syndrome | 553 | Goiter (grades II +) | 116 | Falls | 964 | Natural disasters^(6)^ | 715 | Diabetes mellitus | 2429 |
| LBW + maturity ^(5)^ | 552 | Dental caries | 110 | Anemias ^(3)^ | 947 | COPD ^(10)^ | 616 | Low back pain | 1827 |

(1) Myocardial infarction, angina pectoris, heart failure after myocardial infarction; (2) Respiratory and cardiovascular disorders specific to the perinatal period; (3) Iron deficiency anemia, plus other anemias; (4) Mental disorders due to use of opioids or opioids with multiple drugs; (5) LBW + maturity: Slow fetal growth and fetal malnutrition. LBW: low Birth Weight; (6) Natural disasters including Bam earthquake of 2003; (7) Major depressive disorder; (8) Fetus and newborn affected by maternal factors and by complications of pregnancy, labor and delivery; (9) Upper respiratory tract infections; (10) Chronic Obstructive Pulmonary Disease; (11) Hot flashes & atrophic vaginitis; (12) Bipolar disorders I, II, and cyclothymic disorders
