# Supplementary material for: The burden of disease and injury in Iran 2003
Source: Popul Health Metr. 2009 Jun 15;7:9. doi: 10.1186/1478-7954-7-9 (PMC2711041; doi:10.1186/1478-7954-7-9)
Supplement: Additional file 3 — Detailed comparison of Iranian National Burden of Disease study results with WHO's estimations for EMR-B 2002. We compared Iran's NBD study results with WHO estimates for 2002. Here we describe the differences and conclude about the reasons for their existence. [file 1478-7954-7-9-S3.doc]

**Comparison of Iran’s national burden of disease study results for 2003 with WHO burden of disease estimates for 2002**

We compared Iran’s NBD study results with WHO estimates for 2002. Here we describe the differences and conclude about the reasons for their existence.

**(A) Rationale for choosing the WHO 2002 estimates for comparison**

(1) WHO’s estimates for 2002 are available for world’s regions and sub-regions. Iran resides in sub-region B of WHO’s Eastern Mediterranean Region (EMR) and therefore, we compared Iran’s NBD study burden estimates with those for EMR-B. (2) WHO’s estimates for 2002 are available by DALY, YLL, YLD, age, and sex. (3) Discount rate and age weighing for these estimates are the same as those used in Iran’s NBD study. (4) These estimates are presented along with the pertinent population numbers by sub-region, age, and sex. Therefore, the rates for DALY, YLL, and YLD were calculable for EMR-B and comparable with Iran’s NBD study estimates. (5) These estimates are readily accessible through WHO’s web site [1].

**(B) Comparison of DALY estimates**

DALY estimates by Iran’s NBD study are higher than those for EMR-B (table S1). Comparison of DALYs by age shows that Iran’s NBD study DALY estimates in 0-4 year age group are lower than those for EMR-B, but higher in other age groups, especially in 15-29, 30-44, 70-79, and 80+ years (figure S1).

Table S1: DALY, YLL, and YLD rates in 100000 by sex, Iran 2003 and EMR-B 2002

| **Measure** | **Place** | **Male** | **Female** | **Both Sexes** |
| --- | --- | --- | --- | --- |
| DALY | EMR-B | 17956 | 15743 | 16891 |
|  | Iran | 22715 | 20404 | 21572 |
| YLL | EMR-B | 8951 | 6359 | 7704 |
|  | Iran with Bam* | 10037 | 6527 | 8301 |
|  | Iran without Bam* | 8527 | 5350 | 6956 |
| YLD | EMR-B | 9005 | 9383 | 9187 |
|  | Iran | 12678 | 13878 | 13271 |

* Bam earthquake of 2003 in Southeast Iran

Source: Iran’s NBD study for 2003 and WHO’s GBD study for 2002 [1]

Figure S1: Total DALY rates in 100,000 by age, Iran 2003 and EMR-B 2002

Source: Iran’s NBD study for 2003 and WHO’s GBD study for 2002 [1]

**(C) Comparison of YLL estimates**

Table S1 shows that YLL estimates by Iran’s NBD were higher by those by WHO for EMR-B. The Bam earthquake in Southeast Iran in year 2003, our reference year of calculations, caused about 30000 deaths. The same table shows that the YLL estimate without the mortality from Bam earthquake is lower than WHO’s similar estimate.

Figure S2 shows that the YLL estimates for Iran are higher than WHO’s estimates only in 15-44 year age groups. The main source of this difference is the difference in mortality rates from traffic accidents in the two studies. Traffic accidents mortality rate estimates, which have the greatest differences in these age groups, were 61.6 in 100000 in WHO’s study and 76.6 in 100000 for males in Iran’ NBD study that was estimated based on death registry.

Differences in YLL estimates for other age groups are due to the fact that WHO’s study used a model-based method for estimation of mortality and YLL, whereas Iran’s NBD study used the death registration system. ***Since the methods and values used for standard life expectancy, discount rate, and age weighting were identical in the two studies, the observed differences in YLL estimates are due to difference in source of data for estimation of death rates by age, sex, and cause.***

Figure S2: YLL rates in 100000 by age and sex, Iran 2003

(without Bam earthquake) and EMR-B 2002

Source: Iran’s NBD study for 2003 and WHO’s GBD study for 2002 [1]

**(D) Comparison of YLD estimates**

Table S1 shows that the YLD estimates in Iran’s NBD study are higher by 4000 years per 100000 in average than WHO’s estimates. Age distribution of these estimates shows that Iran’s NBD study estimates are lower in 0-14 year and higher in 15+ age groups, with the highest difference in 80+ years (figure S3). Differences by sex between Iran’s and WHO’s study are near zero in 0-14 year age groups. In 15-60 year age groups, YLD estimates by Iran’s NBD study are higher for females and in 60+ age groups are higher for males (figure S4).

Figure S3: YLD rates in 100000 by age, Iran 2003 and EMR-B 2002

Source: Iran’s NBD study for 2003 and WHO’s GBD study for 2002 [1]

Figure S4: Difference of YLD rates in 100000 between the two studies

by age and sex, Iran 2003 and EMR-B 2002

Source: Iran’s NBD study for 2003 and WHO’s GBD study for 2002 [1]

**(E) Comparison of YLD estimates for GBD clusters**

Figure S5 and table S2 show the YLD estimates by Iran’s NBD study and by WHO for GBD clusters. Iran’s NBD study estimates for noncommunicable diseases are very higher in females and higher in males as compared with WHO estimates. For injuries, Iran’s NBD study estimates are higher in males and lower in females. For GBD’s cluster I, Iran’s NBD study estimates are higher for both sexes. YLD estimates for non communicable diseases in females are higher in 15+ years, with highest differences in 15-59 year age groups. Iran’s NBD study higher YLD estimates for accidents and injuries were much higher in 70+ years.

Figure S5: Difference of YLD rates in 100000 between the two studies

by GBD clusters, Iran 2003 and EMR-B 2002

Cluster I: Communicable, maternal, perinatal, and nutritional conditions;

Cluster II: Noncommunicable diseases; Cluster III: Injuries

Source: Iran’s NBD study for 2003 and WHO’s GBD study for 2002 [1]

Table S2: Differences of YLD rates in 100000 between the two studies

by age, sex, and GBD clusters, Iran 2003 and EMR-B 2002

| **GBD Clusters** | **Age** | **0-4** | **5-14** | **15-29** | **30-44** | **45-59** | **60-69** | **70-79** | **80+** |
| --- | --- | --- | --- | --- | --- | --- | --- | --- | --- |
| **Cluster I: Communicable, maternal, perinatal, and nutritional conditions** | Males | 2170 | 293 | 581 | 362 | 442 | 284 | 235 | 144 |
|  | Females | 386 | 159 | 38 | 203 | 588 | 173 | -31 | -125 |
| **Cluster II:**  **Noncommunicable diseases** | Males | -1642 | -730 | 2736 | 3399 | 1626 | 4027 | 5960 | 4392 |
|  | Females | -1435 | -1185 | 5119 | 8148 | 10086 | 3675 | 4742 | 3004 |
| **Cluster III:**  **Injuries** | Males | -116 | -839 | 755 | 2835 | 1929 | 2230 | 7171 | 24266 |
|  | Female | -255 | -1112 | -475 | 59 | 365 | 1311 | 3783 | 22526 |

Source: Iran’s NBD study for 2003 and WHO’s GBD study for 2002 [1]

**(E1) Comparison of YLD estimates for infectious and parasitic diseases and respiratory infections, maternal, perinatal, and nutritional conditions**

YLD estimate by Iran’s NBD study minus YLD estimate by WHO:

Infectious and parasitic diseases = -111;

Maternal conditions = -137;

Respiratory infections= 151;

Perinatal conditions= 343;

Nutritional deficiencies = 203.

**(E1-1) Comparison of YLD estimates for infectious and parasitic diseases**

For infectious diseases, WHO estimates for YLD from diarrhea are lower than Iran’s NBD study estimates. On the other hand, WHO has estimated a YLD rate of 64 in 100000 for trachoma in Iran, whereas trachoma has entered the control phase in Iran years ago. The situation is similar for schistosomiasis, which is exiting the eradication phase in Iran.

**(E1-2) Comparison of YLD estimates for perinatal conditions**

Table S3 shows the important differences between the YLD estimates for perinatal conditions between the two studies. Source of these differences is that in Iran’s NBD study, the estimates are based on a study by Sodagari [2], which gave direct estimates for perinatal morbidity. Due to the specific disease classification scheme used in GBD, all of these disease conditions fall in the ‘other perinatal conditions’. Therefore, this cause has 307 YLD in 100000 in Iran’s NBD study, but 18 YLD in 100000 in WHO’s study based on residual disease burden estimation. Besides other perinatal diseases, low birth weight YLL estimates are also different. Table S4 demonstrates the differences in YLD estimates for detailed perinatal and childhood causes between Iran’s NBD and WHO’s studies. Direct estimates by Sodagari [2] are shown in this table, whereas WHO’s study has indirect model-based estimates only for a higher categorization level.

Table S3: YLD rates in 100000 for perinatal and childhood diseases, Iran 2003 and EMR-B 2002

| **Condition** | **Iran** | **EMR-B** |
| --- | --- | --- |
| Low birth weight | 176 | 34 |
| Birth asphyxia and birth trauma | 126 | 213 |
| Other perinatal conditions | 307 | 18 |
| Perinatal conditions (total) | 609 | 266 |

Source: Incidence/prevalence in Iran [2,3] and YLD in EMR-B [1]

Table S4: YLD rates in 100000 for detailed perinatal and childhood causes,

Iran 2003 and EMR-B 2002

| **Condition** | **Code** | **Iran** | **EMR- B** |
| --- | --- | --- | --- |
| Term, Small for gestational age | P05 | 26 | * |
| Pre-term, Appropriate for gestational age | P07.1-P07.2 | 91 | * |
| Pre-term, Small for gestational age | P07.3 | 58 | * |
| Low birth weight |  | 176 | 34 |
| Fetus and newborn affected by complications of placenta, cord, and membranes | P02 | 6 | * |
| Birth trauma | P10-P15 | 64 | * |
| Respiratory and cardiovascular disorders specific to the perinatal period | P20-P29 | 54 | * |
| Birth asphyxia and birth trauma |  | 125 | 213 |
| Fetus and newborn affected by maternal factors and by complications of pregnancy, labor and delivery | P00-P04 | 72 | * |
| Infections specified to the perinatal period | P35-P39 | 14 | * |
| Hemorrhagic and hematological disorders of fetus and newborn | P50-P61 | 2 | * |
| Transitory endocrine and metabolic disorders specific to fetus and newborn | P70-P74 | 1 | * |
| Digestive system disorders of fetus and newborn | P75-P78 | 1 | * |
| Conditions involving the integument and temperature regulation of fetus and newborn | P80-P83 | 0 | * |
| Other certain conditions originating in the perinatal period |  | 219 | * |
| Other perinatal conditions |  | 309 | 18 |
| Perinatal conditions (total) |  | 609 | 266 |

* No estimates by WHO at this level of cause categorization details.

Source: Incidence/prevalence in Iran [2,3] and YLD in EMR-B [1]

There are drastic differences between the two studies’ estimates for DALY and its pattern in under-five years’ population. These differences are due to different sources of input data used for estimations of DALYs (table S5). WHO’s sources indicate the highest mortality and morbidity rates in these age groups are for infectious diseases (about 4000 DALYs in 100000 difference for these diseases), whereas the source of Iran’s NBD study shows this to be due to perinatal conditions [2-4]. DALY due to diarrhea and respiratory infections in 0-4 year population in Iran is 3246 and 2535 years respectively in WHO’s study, whereas Iran’s NBD study estimates are 323 and 110 respectively, which are based on Demographic and Health Survey (DHS) [5].

Table S5: DALY rates in 100000 for GBD clusters and groups of causes

in 0-4 year population, Iran 2003 and EMR-B 2002

| **Cluster** | **Condition** | | **EMR-B** | **Iran** | **Difference** |
| --- | --- | --- | --- | --- | --- |
| I. | Communicable, maternal, perinatal and nutritional conditions | | 19181 | 16141 | -3041 |
|  | A. | Infectious and parasitic diseases | 5474 | 703 | -4770 |
|  | B. | Respiratory infections | 2535 | 110 | -2425 |
|  | D. | Perinatal conditions | 9359 | 14764 | 5405 |
|  | E. | Nutritional deficiencies | 1813 | 563 | -1250 |
| II. | Noncommunicable diseases | | 11979 | 6225 | -5755 |
|  | A. | Malignant neoplasms | 256 | 145 | -111 |
|  | E. | Neuropsychiatric conditions | 2852 | 154 | -2698 |
|  | G. | Cardiovascular diseases | 592 | 161 | -430 |
|  | H. | Respiratory diseases | 735 | 362 | -373 |
|  | M. | Congenital anomalies | 5990 | 2575 | -3416 |
| III. | Injuries | | 4204 | 4969 | 765 |
|  | A. | Unintentional injuries | 4085 | 4947 | 862 |

Source: Incidence/prevalence and mortality in Iran [2,4,5] and DALY in EMR-B [1]

**(E1-3) Comparison of YLD estimates for nutritional deficiencies**

The greatest difference in nutritional deficiencies’ YLD estimates is for iron deficiency anemia (table S6). Figures S6 and S7 show this difference along age groups in two sexes, which is primarily due to different age patterns of incidence in the two studies. Source of Iran’s NBD study estimations is the National Study of Micronutrients whose results are valid not only at the national level, but also at the level of 11 climes of Iran [6]. Prevalence rates of iron deficiency anemia used as inputs for estimation with DISMOD are shown in table S7. No direct deaths were assumed for this disease in Iran in 2003 due to access to treatment and hence a mortality rate less than 1 in 100000 was put into DISMOD. Duration of disease was set as constant and equal to three months and disability weights were identical with GBD weights.

Table S6: YLD rates in 100000 for nutritional deficiencies, Iran 2003 and EMR-B 2002

| **Condition** | **Iran** | **EMR-B** |
| --- | --- | --- |
| Nutritional deficiencies | 573 | 370 |
| Protein-energy malnutrition | 26 | 69 |
| Iodine deficiency | 132 | 84 |
| Vitamin A deficiency | 4 | 0 |
| Iron deficiency anemia | 403 | 218 |
| Other nutritional disorders | 8 | 0 |

Source: Incidence/prevalence in Iran [6,7] and YLD in EMR-B [1]

Figure S6: Difference of YLD rates in 100000 between the two studies

for iron deficiency anemia by age in females, Iran 2003 and EMR-B 2002

Source: Incidence/prevalence in Iran [6] and YLD in EMR-B [1]

Figure S7: Difference of YLD rates in 100000 between the two studies

for iron deficiency anemia by age in males, Iran 2003 and EMR-B 2002

Source: Incidence/prevalence in Iran [6] and YLD in EMR-B [1]

Table S7: Prevalence rates in 100000 for iron deficiency anemia

used in Iran’s national burden of disease study for 2003

| **Age (years)** | **Males** | **Females** |
| --- | --- | --- |
| 0-4 | 140.0 | 137.0 |
| 5-14 | 43.8 | 42.3 |
| 15-29 | 30.8 | 82.6 |
| 30-44 | 12.9 | 88.8 |
| 45-59 | 20.5 | 60.5 |
| 60-69 | 21.0 | 37.3 |
| 70-79 | 21.0 | 21.6 |
| 80+ | 21.0 | 21.0 |
| 0+ | 40.8 | 76.4 |

Source: [6]

**(E2) Comparison of YLD estimates for noncommunicable diseases**

The outstanding difference in YLD estimates for noncommunicable disease between the two studies is evident from table S1. This difference is more for females. WHO estimates for noncommunicable diseases in EMR-B are higher than Iran’s NBD study estimates in 0-14 year age groups and lower in 15+ years (figure S8). These differences are highest in 15-59 year females (figure S9). Table S8 shows the noncommunicable diseases specific to females or with higher prevalence in them, for which separate YLL, YLD, and DALY estimations were performed in Iran’s NBD study, but were left in residual diseases in WHO’s study. These diseases increased the YLD for females by 17%, and an important point is that if they are not included, YLD estimates for females by Iran’s NBD study is lower than those for males. Table S9 shows negligible difference between the two studies’ estimates for malignant neoplasms, diabetes mellitus, congenital anomalies, and oral conditions, among other groups with varying degrees of differences.

Figure S8: YLD rates in 100000 for noncommunicable diseases

by age, Iran 2003 and EMR-B 2002

Source: Iran’s NBD study for 2003 and WHO’s GBD study for 2002 [1]

Figure S9: Difference of YLD rates in 100000 between the two studies

for noncommunicable diseases by age and sex, Iran 2003 and EMR-B 2002

Source: Iran’s NBD study for 2003 and WHO’s GBD study for 2002 [1]

Table S8: Noncommunicable diseases for which separate burden estimation was performed

in Iran’s NBD study for 2003 and left in residuals by WHO’s GBD study for 2002

| **Condition** | **Code** |
| --- | --- |
| Menorrhagia duo to leiomyoma and other | D25, N93 |
| Polycystic ovarian syndrome | E28.2 |
| General anxiety disorder | F41.1 |
| Carpal tunnel syndrome | G 56 |
| Tension headache (episode) | G44.2 |
| Mitral valve prolapse | I34.1 |
| Acne vulgaris | L70.0 |
| Acute cystitis | N30.0 |
| Endometriosis | N80 |
| Female genital prolapse | N81 |
| Premenstrual syndrome | N94.3 |
| Menopause (hot flashes & atrophic vaginitis) | N95.1, N95.2 |
| Pregnancy, delivery, abortion, stillbirth | O03-O06, O80-O84 |
| Hyperemesis gravidarum | O21 |
| Pyelonephritis during and after pregnancy | O23.0 |
| Breast abscess and mastitis | O91 |

Source: Incidence/prevalence [4, 8-12]

Table S9: Difference in YLD rates in 100000 between the two studies

for noncommunicable diseases by groups, Iran 2003 and EMR-B 2002

| **Groups** | **Males** | | **Females** | | **Both sexes** | | |
| --- | --- | --- | --- | --- | --- | --- | --- |
|  | **EMR-B** | **Iran** | **EMR-B** | **Iran** | **EMR-B** | **Iran** | **Difference** |
| Malignant neoplasms | 17 | 68 | 40 | 58 | 28 | 63 | 36 |
| Diabetes mellitus | 160 | 139 | 224 | 233 | 191 | 185 | -5 |
| Endocrine and metabolic disorders | 84 | 219 | 86 | 443 | 85 | 330 | 246 |
| Neuropsychiatric conditions | 2620 | 3284 | 2837 | 3978 | 2724 | 3627 | 903 |
| Sense organ diseases | 1355 | 208 | 1612 | 233 | 1479 | 220 | -1258 |
| Cardiovascular diseases | 310 | 592 | 232 | 972 | 273 | 780 | 508 |
| Respiratory diseases | 507 | 589 | 269 | 499 | 392 | 545 | 153 |
| Digestive diseases | 268 | 540 | 331 | 709 | 298 | 623 | 325 |
| Genitourinary diseases | 125 | 704 | 84 | 1144 | 105 | 922 | 817 |
| Skin diseases | 33 | 494 | 45 | 567 | 39 | 530 | 491 |
| Musculoskeletal diseases | 317 | 865 | 390 | 1761 | 352 | 1308 | 956 |
| Congenital anomalies | 221 | 178 | 225 | 189 | 223 | 184 | -39 |
| Oral conditions | 216 | 196 | 221 | 192 | 219 | 194 | -24 |

Source: Iran’s NBD study for 2003 and WHO’s GBD study for 2002 [1]

**(E2-1) Comparison of YLD estimates for endocrine and metabolic diseases**

Iran’s NBD study estimated separate burden for three endocrine and metabolic diseases that were left in residual diseases by WHO’s study, namely congenital hypothyroidism (E03.0, E03.1), phenylketonuria (E70.0, E70.1), and polycystic ovarian syndrome (E28.2). These conditions constitute 80% of YLD for endocrine and metabolic diseases, and cause the difference in YLD estimates for this group between the two studies.

**(E2-2) Comparison of YLD estimates for neuropsychiatric diseases**

Table S10 shows the differences for neuropsychiatric diseases’ YLD estimates between the two studies, with both considerable and negligible differences. Separate estimations for insomnia, mental retardation, and alcohol abuse disorders were not performed in Iran’s NBD study. Figure S10 shows the YLD estimates for drug use disorders in Iran’s NBD study, which includes only opium, its derivatives, and cannabis. This figure demonstrates that the incidence rates and their age patterns are different for these disorders between the two studies. Table S11 shows the prevalence and mortality rates for drug use disorders used as inputs for estimation with DISMOD and their source studies.

Table S10: Difference in YLD rates in 100000 between the two studies

for neuropsychiatric disease, Iran 2003 and EMR-B 2002

| **Condition** | **Difference** |
| --- | --- |
| Drug use disorders | 453 |
| Schizophrenia | -149 |
| Bipolar disorders | 101 |
| Panic disorder | 64 |
| Epilepsy | 42 |
| Obsessive-compulsive disorder | 31 |
| Unipolar depressive disorders | -31 |
| Post-traumatic stress disorder | 27 |
| Alzheimer and Other dementias | 23 |
| Multiple sclerosis | 14 |
| Parkinson’s disease | 10 |
| Migraine | 10 |

Source: Incidence/prevalence in Iran [8,13] and YLD in EMR-B [1]

Figure S10: YLD rates in 100000 for drug use disorders by age, Iran 2003 and EMR-B 2002

Source: Incidence/prevalence in Iran [8,13] and YLD in EMR-B [1]

Table S11: Prevalence and mortality rates in 100000 for drug use disorders

used in Iran’s national burden of disease study for 2003

| **Age** | **Males** | | **Females** | |
| --- | --- | --- | --- | --- |
|  | **Prevalence** | **Mortality** | **Prevalence** | **Mortality** |
| 0-4 | 0.0000 | 0.0010 | 0.0000 | 0.0005 |
| 5-14 | 0.0000 | 0.0002 | 0.0000 | 0.0002 |
| 15-29 | 52.3979 | 0.0521 | 3.2454 | 0.0014 |
| 30-44 | 65.6303 | 0.1527 | 5.3436 | 0.0064 |
| 45-59 | 29.5984 | 0.1484 | 11.8648 | 0.0063 |
| 60-69 | 23.288 | 0.0480 | 12.1091 | 0.0055 |
| 70-79 | 9.9056 | 0.0436 | 17.9263 | 0.0190 |
| 80+ | 6.7000 | 2.1469 | 31.6000 | 0.0884 |
| 0+ | 33.904 | 0.0755 | 4.3373 | 0.0035 |

Source: [4,8,13]

Figures S11 to S14 demonstrate the differences in age patterns of prevalence and incidence of bipolar disorders, panic disorder, epilepsy, and obsessive-compulsive disorder between the two studies. Figures S15 and S16 show that in spite of differences in prevalence rates of unipolar depressive disorder and schizophrenia in data sources used in the two studies, the age patterns are similar.

Figure S11: YLD rates in 100000 for bipolar disorders by age, Iran 2003 and EMR-B 2002

Source: Incidence/prevalence in Iran [8] and YLD in EMR-B [1]

Figure S12: YLD rates in 100000 for panic disorder by age, Iran 2003 and EMR-B 2002

Source: Incidence/prevalence in Iran [8] and YLD in EMR-B [1]

Figure S13: YLD rates in 100000 for epilepsy by age, Iran 2003 and EMR-B 2002

Source: Incidence/prevalence in Iran [8] and YLD in EMR-B [1]

Figure S14: YLD rates in 100000 for obsessive-compulsive disorder

by age, Iran 2003 and EMR-B 2002

Source: Incidence/prevalence in Iran [8] and YLD in EMR-B [1]

Figure S15: YLD rates in 100000 for unipolar depressive disorder

by age, Iran 2003 and EMR-B 2002

Source: Incidence/prevalence in Iran [8] and YLD in EMR-B [1]

Figure S16: YLD rates in 100000 for schizophrenia by age, Iran 2003 and EMR-B 2002

Source: Incidence/prevalence in Iran [8] and YLD in EMR-B [1]

**(E2-3) Comparison of YLD estimates for cardiovascular diseases**

Whereas incidence, prevalence, and age pattern of ischemic heart diseases differ in the two studies (figure S17), for cerebrovascluar diseases the age patterns are similar, and the incidence rates are different (figure S18).

Figure S17: YLD rates in 100000 for ischemic heart diseases by age, Iran 2003 and EMR-B 2002

Source: Incidence/prevalence in Iran [14,15] and YLD in EMR-B [1]

Figure S18: YLD rates in 100000 for cerebrovascular diseases by age, Iran 2003 and EMR-B 2002

Source: Incidence/prevalence in Iran [14,15] and YLD in EMR-B [1]

**(E2-4) Comparison of YLD estimates for musculoskeletal diseases**

Besides the items in GBD cause list, some other conditions were included in Iran’s NBD study that fall into ‘other musculoskeletal diseases’, namely neck arthrosis, systemic lupus erythematosus, frozen shoulder, rotator cuff syndrome, and elbow epicondylitis. There was not a valid source for estimation disease transition rates for gout in Iran. All the input data for disease modeling in this group were taken from data bank of Community Oriented Program for Control of Rheumatic Diseases (COPCORD) national study performed in 2001 [10]. Table S12 shows the age pattern of prevalence from COPCORD study used as input for disease modeling with DISMOD for knee arthrosis and low back pain. No mortality and no remission were included for unilateral or bilateral knee arthrosis and the periodic remissions and exacerbation periods were accounted for in calculation of disability weights. Mean disease duration was estimated as 2-3 weeks for low back pain without sciatica, and no deaths included for this disease. For low back pain with sciatica, mean duration was four weeks with 85% remission and six weeks with 100% remission and with no deaths included. Disability weights for these two diseases were equal and the same as those used in GBD. Figures S19 and S20 show the differences in incidence rates and age patterns for these two disease between Iran’s NBD study and WHO’s study.

Table S12: Prevalence in 100000 from COPCORD study used as inputs

for modeling of knee arthrosis and low back pain

| **Age** | **Arthrosis of knee, unilateral** | | **Arthrosis of knee, bilateral** | | **Low back pain, without sciatica** | | **Low back pain, with sciatica** | |
| --- | --- | --- | --- | --- | --- | --- | --- | --- |
| Sex | Males | Females | Males | Females | Males | Females | Males | Females |
| 0-4 | 0.0000 | 0.0000 | 0.0000 | 0.0000 | 0.0000 | 0.0000 | 0.0000 | 0.0000 |
| 5-14 | 0.0000 | 0.0000 | 0.0000 | 0.0000 | 0.0000 | 0.0000 | 0.0000 | 0.0000 |
| 15-29 | 2.8403 | 2.2478 | 1.8219 | 3.0746 | 42.3519 | 97.2236 | 3.0269 | 2.5113 |
| 30-44 | 22.5861 | 38.1007 | 52.4475 | 102.5639 | 118.9881 | 211.868 | 7.298 | 14.2411 |
| 45-59 | 56.2359 | 94.4771 | 115.2961 | 283.366 | 125.2176 | 233.0129 | 13.1523 | 15.7381 |
| 60-69 | 85.044 | 129.683 | 240.4692 | 489.9135 | 158.3578 | 233.4294 | 2.9326 | 8.6455 |
| 70-79 | 94.1176 | 128 | 384.3137 | 604 | 203.9216 | 308 | 0 | 4 |
| 80+ | 94.1176 | 128 | 384.3137 | 604 | 203.9216 | 308 | 0 | 4 |
| 0+ | 16.7323 | 26.0998 | 42.0133 | 84.5334 | 61.1134 | 115.1018 | 3.8381 | 5.6435 |

Source: [10]

Figure S19: YLD rates in 100000 for knee osteoarthritis by age, Iran 2003 and EMR-B 2002

Source: Incidence/prevalence in Iran [10] and YLD in EMR-B [1]

Figure S20: YLD rates in 100000 for low back pain by age, Iran 2003 and EMR-B 2002

Source: Incidence/prevalence in Iran [10] and YLD in EMR-B [1]

**(E3) Comparison of YLD estimates for unintentional injuries**

Figure S21 shows the differences in YLD for unintentional injuries in males between the two studies. Figure S22 shows that for both sexes together, the greatest differences are for 60+ age groups. Figure S23 for road traffic accidents shows that the differences between the two studies are not only in incidence rates estimates but also in age patterns as well. Figure S24 for falls shows WHO’s underestimates for falls’ YLD in EMR-B and not only the role of osteoporosis is not accounted for in increasing the YLD in older ages, but also it seems that the higher prevalence of osteoporosis in the elderly helps them mysteriously to tolerate less YLD rates than young and middle-aged population of EMR-B.

Table S13 demonstrates the estimated incidence rates of selected unintentional injuries leading to hospitalization in Iran in 2003 [16] which was performed on public and private hospitals of different provinces including all hospitalization cases due to injuries and their results used for estimation of injuries’ YLD. Indeed Iran’s NBD study estimates for YLD of these diseases differ from those by WHO, due to differences in incidence rates and their age patterns. Iran’s NBD study results demonstrate that the estimates of falls’ YLD in elderly influence the total value of their YLD. WHO’s estimates for YLD in elderly seem to be lower than the actual values since the role of osteoporosis in increasing YLD in these age groups is not accounted for properly - and not only for falls, but also for other accidents such as road traffic accidents in pedestrians and other accidents due to mechanical forces. Fragility of the elderly results in femoral neck fractures or distal radius (Colles’) fractures (ICD-10 S52.5) with mechanical forces less than what would cause a fracture in the young and middle-aged population. Therefore, even trivial falls in elderly may lead to femoral neck fracture with more severe and lengthy disability and even mortality as compared with younger age groups. Low access to and limited affordability for expensive services of hip replacement arthroplasty contribute to elongation of the resultant disability duration in developing countries. These lead to higher YLD rates for accidents in the elderly, but the absolute value of their YLD is lower than that for young and middle-aged population in countries like Iran where the proportion of elderly population is still not high. Figure S25 shows that in accidents and injuries like burns in which osteoporosis does not aggravate the disability, Iran’s NBD study estimates for YLD are similar with WHO’s estimates in terms of age pattern, although there are differences in incidence rates.

Figure S21: YLD rates in 100000 for unintentional injuries by sex, Iran 2003 and EMR-B 2002

Source: Incidence/prevalence in Iran [16-18] and YLD in EMR-B [1]

Figure S22: YLD rates in 100000 for unintentional injuries by age, Iran 2003 and EMR-B 2002

Source: Incidence/prevalence in Iran [16-18] and YLD in EMR-B [1]

Figure S23: YLD rates in 100000 for road traffic accidents

by age and sex, Iran 2003 and EMR-B 2002

Source: Incidence/prevalence in Iran [16] and YLD in EMR-B [1]

Figure S24: YLD rates in 100000 for falls by age, Iran 2003 and EMR-B 2002

Source: Incidence/prevalence in Iran [16] and YLD in EMR-B [1]

Table S13: Incidence rates in 100000 for selected unintentional injuries

leading to hospitalization in Iran used as inputs for disease modeling

| **Condition** | **Unintentional injuries (total)** | | **Road traffic accidents** | | **Burns** | | **Falls** | |
| --- | --- | --- | --- | --- | --- | --- | --- | --- |
| Age | Inpatient | Outpatient | Inpatient | Outpatient | Inpatient | Outpatient | Inpatient | Outpatient |
| 0 | 436 | 1627 | 67 | 186 | 74 | 415 | 149 | 565 |
| 1-4 | 525 | 2377 | 101 | 200 | 62 | 371 | 182 | 860 |
| 5-14 | 423 | 1599 | 160 | 380 | 13 | 99 | 155 | 480 |
| 15-24 | 970 | 3149 | 568 | 1213 | 22 | 105 | 155 | 539 |
| 25-34 | 790 | 2644 | 438 | 915 | 24 | 101 | 128 | 384 |
| 35-44 | 635 | 2150 | 350 | 574 | 16 | 111 | 121 | 419 |
| 45-54 | 743 | 2177 | 392 | 618 | 18 | 105 | 182 | 482 |
| 55-64 | 772 | 1752 | 391 | 535 | 23 | 94 | 231 | 534 |
| 65-74 | 779 | 1388 | 297 | 431 | 15 | 39 | 360 | 541 |
| 75-84 | 1089 | 1454 | 330 | 356 | 20 | 53 | 646 | 621 |
| 85+ | 1426 | 2165 | 212 | 601 | 34 | 120 | 1083 | 1083 |
| 0+ | 718 | 2321 | 360 | 711 | 23 | 123 | 171 | 507 |

Source: [16-18]

Figure S25: YLD rates in 100000 for burns by age and sex, Iran 2003 and EMR-B 2002

Source: Incidence/prevalence in Iran [16] and YLD in EMR-B [1]

**(F) Conclusion form comparisons with WHO estimates for EMR-B in 2002**

Partitioning analysis of the differences between Iran’s NBD study higher estimates for DALY rates in Iran and the lower similar estimates by WHO for EMR-B reveals the following:

**(1)** In terms of age, the DALY difference resides mainly in the 15-44 and 70+ age groups.

**(2)** In terms of YLL, the difference returns to the mortality from Bam earthquake of 2003. Iran’s NBD study YLL estimate without the Bam earthquake is even lower. Iran’s NBD study estimates for YLL rates form road traffic accidents are higher but this does not lead to surplus in all cause YLL rate without Bam.

**(3)** The main part of difference is due to Iran’s NBD study higher YLD rate estimates in 15+ age groups, which are most pronounced in 80+ years. The difference is also higher for females in 15-59 and for males in 60+ years. While the disability weights and durations are identical with those used in the GBD study, the discovered differences in YLD rates originate from (a) independent YLD estimation for causes that are included in Iran’s NBD study but not in the GBD, and (b) differences in age patterns and incidence rates of causes that are included both in GBD and Iran’s NBD study.

**(3a)** Iran’s NBD study estimated the YLD (and YLL and DALY) rates for a number of causes added to the GBD list cause, for which the YLD estimates fall within the residual categories in WHO’s study. Their YLD estimates within the residual categories are almost lower than their separate YLD estimates. These added causes include some of perinatal conditions, endocrine and metabolic diseases, and conditions specific to or with higher prevalence in females.

**(3b)** Differences in age patterns of causes are exemplary for conditions such as iron deficiency anemia, ischemic heart diseases, drug use disorders, bipolar disorders, and panic disorder. Different incidence rates and age patterns of some causes were based on results of national studies in Iran, some of them indicating higher incidences like for drug use disorders, low back pain, osteoarthritis, and obsessive-compulsive disorder. For some other diseases on the contrary, results of national studies showed markedly lower prevalence, like for diarrhea, respiratory inactions, and schizophrenia.

Input data adopted by WHO for modeling injuries’ epidemiology and estimating their YLL and YLD rates in Iran for 2002 lead to results that are considerably different from Iran’s NBD study results, which are based on input data from two national-level studies of injuries epidemiology. These differences are more pronounced for nonfatal outcomes of road traffic injuries, falls, and injuries due to mechanical forces (the latter reside in ‘other injuries’ category in WHO’s GBD study for 2002). WHO’s model underestimates the incidence of non-severe accidents that result in durable and severe disability in old age groups. Such underestimation in turn results in lower values for YLD estimates in elderly. The aggravating role of osteoporosis on such disability could be more appropriately accounted for WHO’s estimates of YLD in the elderly in Iran.

**References**

1. World Health Organization. Revised Global Burden of Disease (GBD) 2002 Estimates. Estimates by WHO Region and Sub-region. Disability adjusted life years (DALY). [http://www.who.int/healthinfo/bodgbd2002revised/en/index.html]

2. Sodagari B. Study on influencing factors on perinatal and neonatal mortality and morbidity in Gilan and West Azarbaijan. Family Health and Population Office, Health Deputy, Health Deputy, Ministry of Health and Medical Education. 2001.

3. Sheikholeslam R, Naghavi M. Estimation of preterm and low birth weight rates in Iran. 2004.

4. Naghavi M: Profile of Death in 23 Provinces of Iran in Year 2003. Tehran: Ministry of Health and Medical Education; 2005.

5. Ministry of Health and Medical Education, in collaboration with Statistical Center of Iran, Civil Registration Organization of Iran, United Nations Children’s Fund (UNICEF), and United Nations Population Fund (UNFPA): Profile of Population and Health, Demographic and Health Survey (DHS). Tehran; 2000.

6. Ministry of Health and Medical Education: National Study of Micronutrients. Tehran; 2001.

7. Ministry of Health and Medical Education: National program of neonatal screening for congenital hypothyroidism. Tehran: 2005.

8. Mohammadi MR, Davidian H, Noorbala AA, Malekafzali H, Naghavi HR, Pouretemad HR, Yazdi SA, Rahgozar M, Alaghebandrad J, Amini H, Razzaghi EM, Mesgarpour B, Soori H, Mohammadi M, Ghanizadeh A: An epidemiological survey of psychiatric disorders in Iran. Clin Pract Epidemol Ment Health 2005 Sep 26;1:16.

9. Delavar B, Azemikhah A, Eslami M: Integrated Monitoring and Evaluation System for Reproductive Health Programs. Tehran: Ministry of Health and Medical Education; 2005.

10. Tehran University of Medical Sciences: Community Oriented Program for Control of Rheumatic Diseases (COPCORD). Tehran; 2001.

11. Toosi P, et al. Comprehensive textbook of skin diseases in Iran. Tehran: Teymurzadeh Publishers. 2001.

12. Aghsaa MM, Ghotbi R. Premenstrual syndrome. Iranian Journal of Current Medicine. 2004, No. 5, 1-3.

13. Yasami M, Shahmohammadi D, Naghavi M: Epidemiologic Study of Drug Abuse in Iran. Tehran: Ministry of Health and Medical Education; 2003.

14. Hormozgan University of Medical Sciences: Persian Gulf Healthy Heart Study. Bushehr; 2001.

15. Isfahan University of Medical Sciences: Isfahan Healthy Heart Study. Isfahan; 2003.

16. Naghavi M, Jafari N, Alaeddini F, Akbari ME. Epidemiology of injuries due to external causes in Iran. Tehran: Ministry of Health and Medical Education; 2003.

17. Ghodsi M, et al. Evaluation of diagnostic and therapeutic interventions and outcomes for patients injured in Bam earthquake and referred to hospitals of Tehran University of Medical Sciences. Trauma and Surgery Research Center, Sina Hospital, Tehran University of Medical Sciences. 2003

18. Ministry of Health and Medical Education. Database of patients referred to Hospitals in Bam and Kerman after Bam earthquake. 2003.
